# Association between pneumonia hospitalisation and long-term risk of cardiovascular disease in Chinese adults: A prospective cohort study

**DOI:** 10.1016/j.eclinm.2022.101761

**Published:** 2022-12-02

**Authors:** Yizhen Hu, Zhijia Sun, Canqing Yu, Yu Guo, Pei Pei, Ling Yang, Yiping Chen, Huaidong Du, Dianjianyi Sun, Yuanjie Pang, Xiaocao Tian, Simon Gilbert, Daniel Avery, Junshi Chen, Zhengming Chen, Liming Li, Jun Lv, China Kadoorie Biobank Collaborative Group

**Affiliations:** a Department of Epidemiology & Biostatistics, School of Public Health, Peking University, Xueyuan Road, Haidian District, Beijing 100191, China; b Peking University Center for Public Health and Epidemic Preparedness and Response, Beijing 100191, China; c Fuwai Hospital Chinese Academy of Medical Sciences, Beijing 100037, China; d Medical Research Council Population Health Research Unit at the University of Oxford, Oxford OX3 7LF, UK; e Clinical Trial Service Unit and Epidemiological Studies Unit, Nuffield Department of Population Health, University of Oxford, Oxford OX3 7LF, UK; f NCDs Prevention and Control Department, Qingdao Center for Disease Control and Prevention, Shandong 266033, China; g China National Center for Food Safety Risk Assessment, Beijing 100022, China

**Keywords:** Pneumonia, Ischaemic heart disease, Arrhythmia, Ischaemic stroke, Hemorrhagic stroke, Heart failure, Risk, Long-term, Short-term, Cohort

## Abstract

**Background:**

Lower respiratory tract infections, including pneumonia, have been associated with short-term increased risk of cardiovascular disease (CVD). However, there is only limited evidence about the long-term impact of pneumonia on the cardiovascular system beyond one year.

**Methods:**

We conducted a prospective matched cohort study based on data from the China Kadoorie Biobank study of 482,017 adults who were enrolled between June 25, 2004, and July 15, 2008, and were free of CVD at baseline and before pneumonia hospitalization. A total of 24,060 patients hospitalised with pneumonia were identified until December 31, 2018, and were matched on age, sex, urban or rural areas, and decile of the frailty index to 223,875 controls. We used the piecewise Cox proportional hazards model to estimate the hazard ratios (HRs) and their 95% confidence intervals (CIs) for pre-specified incident CVD outcomes, including ischaemic heart disease (IHD), arrhythmia, heart failure (HF), ischaemic stroke (IS), and hemorrhagic stroke (HS), at various time intervals through 10 years after pneumonia hospitalization.

**Findings:**

Of the 247,935 pneumonia cases and controls included, the mean age (standard deviation) was 53.5 (10.4), and 40.8% (101,159) were men. During follow-up, 2389 (9.9%) pneumonia cases developed IHD, 489 (2.0%) cases developed arrhythmia, 545 (2.3%) cases developed HF, 1764 (7.3%) cases developed IS, and 348 (1.4%) cases developed HS. After adjustment for sociodemographic characteristics, lifestyle factors, health status and medication, underlying conditions, and family history of CVD, the elevated CVD risk was highest within the first 30 days after pneumonia hospitalisation, with subsequent risk reductions varying by subtypes. The elevated risk remained until the eighth year after pneumonia hospitalisation for IHD, arrhythmia, and HF, with HRs (95% CIs) of 1.48 (1.13–1.93), 2.69 (1.70–4.25), and 4.36 (2.86–6.64), respectively. The risk of stroke associated with pneumonia hospitalisation remained elevated until the seventh year for IS (HR = 1.30; 95% CI: 1.04–1.63), and until the second year for HS (1.39; 1.07–1.80). The above associations were consistently observed across various characteristics of the participants.

**Interpretation:**

In middle-aged and older Chinese adults, pneumonia hospitalisation was associated with short- and long-term CVD risk, with the elevated risk of certain CVD outcomes persisting for up to 8 years.

**Funding:**

10.13039/501100001809National Natural Science Foundation of China, the 10.13039/501100012166National Key R&D Program of China, the 10.13039/501100002855Chinese Ministry of Science and Technology, the Kadoorie Charitable Foundation in Hong Kong, the UK 10.13039/100010269Wellcome Trust.

**Research in context**

**Evidence before this study**

We searched PubMed and Google Scholar for articles published before September 20, 2022, using a combination of terms: (“pneumonia” OR “respiratory infection”) AND (“cardiovascular disease” OR “heart disease” OR “cardiac disease” OR “arrhythmia” OR “cerebrovascular disease” OR “stroke” OR “heart failure”). We manually searched reference lists and retrieved articles as well. Only four cohort studies conducted in Western populations have examined the long-term risk of cardiovascular disease (CVD) risk after pneumonia. These studies had different outcomes, either a certain single CVD outcome, or a composite endpoint, making it difficult to compare their findings directly. Furthermore, the associations of pneumonia with the risk of arrhythmia and hemorrhagic stroke have not been investigated.

**Added value of this study**

In this matched cohort study nested within the China Kadoorie Biobank, we found that pneumonia hospitalisation was associated with an increased risk of ischaemic heart disease, arrhythmia, heart failure, ischaemic stroke, and hemorrhagic strok. Differences existed in the strength of association and duration of elevated risk among five CVD subtypes. The associations were consistently observed across various characteristics of the participants. To the best of our knowledge, the present study is the first large matched cohort study that examined the associations of pneumonia hospitalisation with the long-term risk of five CVD subtypes using the same analysis strategy.

**Implications of all the available evidence**

In recent years, the long-term cardiovascular consequences of coronavirus disease 2019 have become a public health concern. The results of our study suggest that pneumonia caused by pathogens other than severe acute respiratory syndrome coronavirus 2 was associated with short- and long-term CVD risk, with an elevated risk of certain CVD outcomes persisting for up to 8 years. Our findings underscore the importance of prevention of all-cause pneumonia and particular attention to short- and long-term cardiovascular health of patients recovered from pneumonia.

## Introduction

Lower respiratory tract infections, including pneumonia, are the leading infectious cause of death worldwide, with an estimated 2.6 million deaths in 2017.^1^ Although pneumonia is traditionally regarded as a disease confined to the lungs, growing evidence suggests that pneumonia may have a negative impact on multiple organ systems, including the cardiovascular system.^2^^,^^3^

Previous studies found that respiratory infections were associated with increased short-term risk of cardiovascular disease (CVD).^3^^,^^4^ These studies were mainly conducted in the Western populations and focused on CVD risk within three months after infection, using self-controlled case series (SCCS) or case-crossover designs. However, elevated hemostasis or cardiovascular biomarkers have been observed among individuals who had clinically recovered from pneumonia and were associated with increased CVD risk up to one year after pneumonia hospitalisation.^5^^,^^6^ Our recent study, based on the China Kadoorie Biobank (CKB) and using SCCS design, found that the risk remained elevated up to one year after pneumonia hospitalisation for ischaemic heart disease (IHD) and three months for ischaemic stroke (IS).^7^

Only four cohort studies conducted in Western populations have examined the long-term risk of CVD after pneumonia and revealed that pneumonia was associated with an increased risk of IHD, stroke, or heart failure (HF) for up to 10 years.^8^, ^9^, ^10^, ^11^ However, these studies had different outcomes, either a certain single CVD outcome,^9^^,^^10^ or a composite endpoint,^8^ making it difficult to compare their findings directly. Arrhythmia is also common among patients hospitalised with pneumonia^12^; however, no previous study has examined the association between pneumonia hospitalisation and the subsequent risk of arrhythmia. Given a lower incidence of hemorrhagic stroke (HS) in the Western than in the Chinese populations, the specific association of pneumonia with HS risk, which might possess a distinct underlying mechanism to that of IS remained largely unclear. Besides, the relative importance of pneumonia in predicting the CVD risk compared with traditional risk factors for CVD is unknown.

Based on the CKB cohort of 0.5 million Chinese adults, we aimed (1) to examine the associations of pneumonia hospitalisation with the risk of IHD, arrhythmia, HF, IS, and HS over the subsequent 10 years, and how the strength of the associations changed over time; (2) to examine whether the associations were consistent across subpopulations; and (3) to compare the relative importance of pneumonia hospitalisation with traditional CVD risk factors in terms of predicting the CVD risk over 10 years.

## Methods

### Study population

The details of the design and implementation of the CKB have been described.^13^^,^^14^ Briefly, 512,723 participants aged 30–79 years were enrolled between June 25, 2004, and July 15, 2008, from five urban and five rural areas of China and followed up for nearly 15 years by the December 31, 2018. After signing a written informed consent, all participants completed an interviewer-administered questionnaire, physical measurements, and blood sample collection at baseline. Following completion of the baseline survey, three resurveys were conducted in 2008, 2013–2014, and 2020–2021 in randomly selected subsamples of cohort members under similar procedures.^14^ The vital status of the participants was ascertained through the official Disease Surveillance Point death registries.^15^ The disease incidence and hospitalisation events were ascertained by electronic linkage, via a unique personal identification number, to established registries of major diseases (including CVD, cancer, and diabetes) and health insurance (HI) claim database. All events were reviewed, integrated centrally, and standardized using the 10th revision of the International Classification of Diseases codes (ICD-10) by trained staff blinded to the baseline information. The Ethical Review Committee of the Chinese Centre for Disease Control and Prevention (Beijing, China) and the Oxford Tropical Research Ethics Committee, University of Oxford (UK) approved the study. This manuscript conforms to the STROBE reporting guidelines.

### Ascertainment of exposure and outcomes

We identified participants hospitalised with pneumonia during follow-up by extracting medical records that had a discharge diagnosis of pneumonia (ICD-10 codes J12-J18) from the HI claim data. The primary outcomes, ascertained through death and disease registries and HI claim data, were incident IHD (codes I20-I25), arrhythmia (codes I44-I49), HF (codes I50), IS (codes I63), and HS (codes I61). The secondary outcomes were the subtypes of IHD and IS. For IHD, we classified it into major coronary events [fatal IHD and nonfatal myocardial infarction (codes I21-I23), MCE] and IHD other than MCE. For IS, we classified it into lacunar infarction (LACI) and non-lacunar infarction (non-LACI). LACI was small cerebral ischaemic lesions <15 mm in diameter on the brain imaging, whereas non-LACI was any type of IS other than LACI. For information on the ongoing outcome adjudication process of cardiovascular events, please see the Supplementary Methods.

### Selection of the analysis cohort

**Fig. 1:**
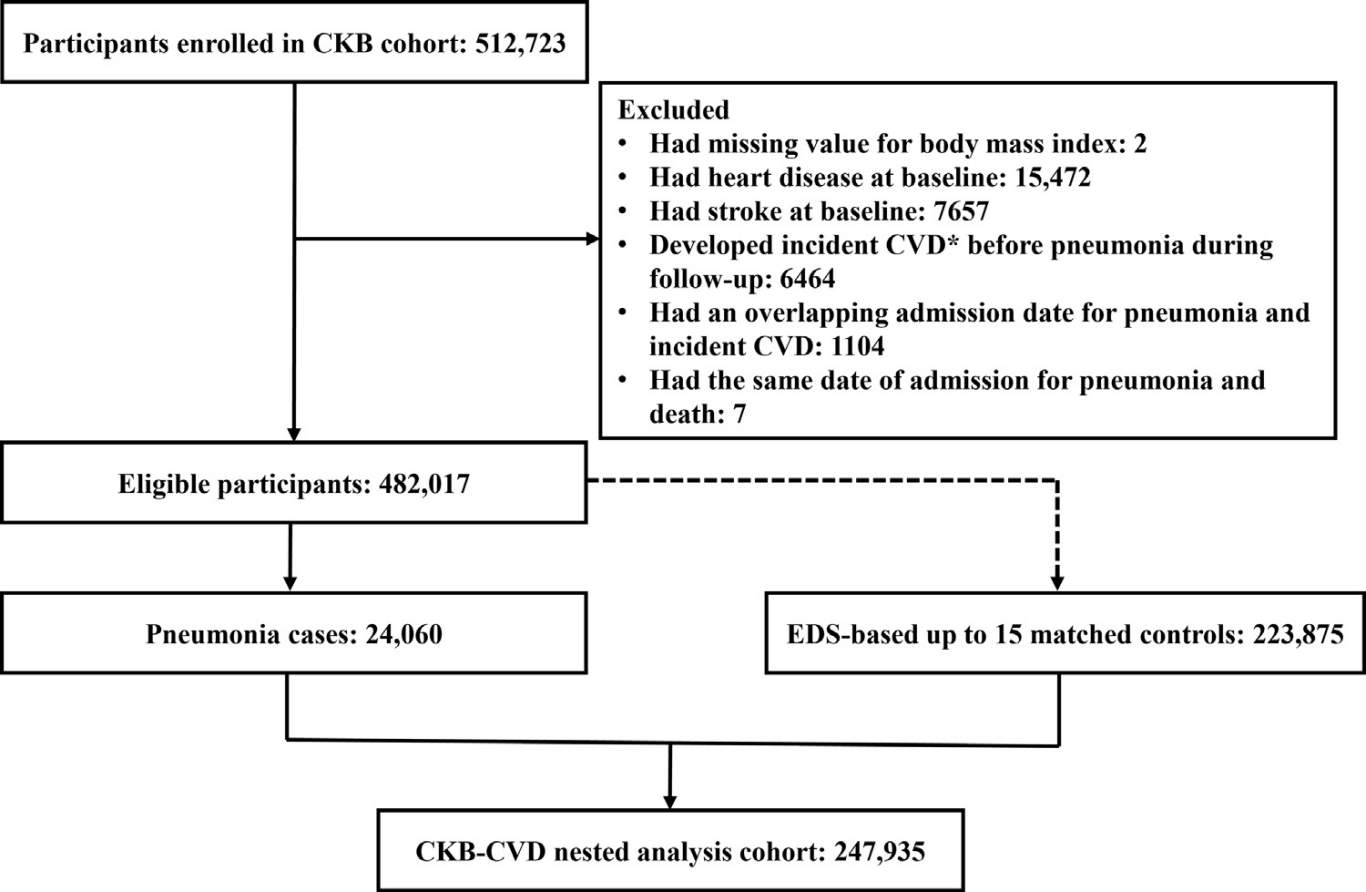
**Selection of the nested analysis cohort from the CKB cohort.** CKB, China Kadoorie Biobank; EDS, exposure density sampling; CVD, cardiovascular disease. ∗The composite endpoint used for exposure density sampling was incident cardiovascular disease, including ischaemic heart disease, arrhythmia, heart failure, ischaemic stroke, and hemorrhagic stroke.

We excluded the following participants sequentially (Fig. 1): (1) those with missing value for body mass index (BMI, n = 2); (2) those who reported previous doctor-diagnosed heart disease (n = 15,472) or stroke (n = 7657) at baseline; (3) those who developed any one of the five primary outcomes before pneumonia hospitalisation during follow-up (n = 6464); (4) those with an overlapping admission date for pneumonia and the first occurrence of the five primary outcomes (n = 1104); and (5) those with the same date of admission for pneumonia and death (n = 7). Among the remaining eligible participants (n = 482,017), we used an “exposure density sampling (EDS)” approach^16^^,^^17^ to build a nested cohort of participants with and without pneumonia hospitalisation. For details of the EDS approach, please see the Supplementary Methods. Briefly, a total of 24,060 participants hospitalised for pneumonia were included in the matching process. For each exposed individual, up to 15 matched controls who were still at risk for incident CVD and still non-exposed at the time at which the exposed one was hospitalised for pneumonia (i.e., the index date) were selected from the risk set. Besides matching for time to exposure, other matched factors included baseline age groups with 5-year increments, sex, urban or rural area, and decile of the frailty index.^18^ The sampling of controls was carried out with replacement to derive unbiased estimators. A sampled control could also be hospitalised with pneumonia and become exposed later. For such controls, the first sampling time was the entry time, and the exposure was included as a time-dependent variable.^17^

### Assessment of covariates

The baseline questionnaire inquired information on sociodemographic characteristics (age, sex, urban or rural residence, marital status, educational attainment), lifestyle factors (tobacco smoking, alcohol consumption, physical activity, and dietary habits), personal and family medical history, self-reported health status, and medication (using antihypertensive or glucose-lowering drugs). Trained staff measured height and weight (used for calculating BMI), waist circumference, blood pressure, lung function, and random plasma glucose.

We defined a family history of CVD as at least one first-degree relative (biological father, mother, and siblings) with heart disease or stroke. The frailty index used in the present analysis was constructed based on 28 self-reported or measured variables at baseline and calculated as the number of deficits present in a person divided by 28 variables considered, ranging from 0.00 to 1.00. More details are available in our previous publication.^18^ The diseases of interest in the present study include hypertension, diabetes, chronic obstructive pulmonary disease (COPD), tuberculosis, asthma, chronic kidney disease, cirrhosis/chronic hepatitis, peptic ulcer, gallstone disease, rheumatoid arthritis, fracture, and cancer, all of which we asked participants whether they have had at baseline. Prevalent hypertension, diabetes, and COPD at baseline were defined based on both self-reported medical diagnosis and baseline measurements.^19^^,^^20^ The status of the 12 abovementioned major underlying conditions was also updated through linkage to the disease registry and HI claim database until the index date.

### Statistical analysis

In the present analyses, the follow-up time was determined from the index date to the date of death, diagnosis of CVD outcomes, loss to follow-up, 10 years after the index date, or December 31, 2018, whichever occurred first. As indicated by previous studies, the increased risk of CVD was maximal shortly after respiratory infections and decreased with time.^7^^,^^8^ We tested the proportional hazard assumption using Schoenfeld residuals and log (−log) plots, suggesting nonproportional hazards for five primary outcomes. Therefore, we applied the piecewise Cox proportional hazards model that allows the hazard ratios (HRs) and their 95% confidence intervals (CIs) of pneumonia hospitalisation on CVD risk to vary over time periods.^21^ In the piecewise Cox proportional hazards model, we grouped the periods after the index date into days 0–30, days 31–90, day 91 to one year, and then yearly up to year 10 according to the previous studies.^7^^,^^8^ The proportional hazard assumption for all outcomes were tested and met for each time period. Owing to small sample sizes in some time periods, we merged periods after the index date into days 0–30, days 31–90, day 91 to one year, years 1–3, years 3–8, and years 8–10 in some subsequent analyses. Time periods after one year in our study are left-open and right-closed.

Multivariable models were jointly stratified by age at index date in five-year interval and study area. The frailty index was also adjusted as a continuous variable to control for any residual confounding not accounted for in the matching process. Detailed information on adjusted covariates was listed in Supplementary Methods. To understand possible temporal changes in some covariates, we compared the risk level of lifestyle factors and medication use between 2004–08 baseline and 2013–14 resurvey by disease status among 23,686 participants who participated in both surveys. Several sensitivity analyses were performed to check the robustness of our findings. Considering the potential effect of recurrent pneumonia episodes on the risk of incident CVD, we repeated our primary analysis by excluding participants who had multiple pneumonia hospitalisations before the incidence of CVD. Since frail people are at high risk for pneumonia hospitalisation, and frailty was also associated with increased CVD risk,^22^ frailty status may confound our findings. We, therefore, restricted the analysis to the robust participants whose frailty index was ≤0.10. In EDS, the follow-up time was counted not from baseline but from a later time. To avoid potential bias introduced by delay-entry, we restricted the exposed group to participants who were hospitalised with pneumonia in the first five years of follow-up and re-performed the control sampling process. To exclude the potential impact of hospital-acquired pneumonia, we restricted the exposed group to participants without any other hospital admission in the previous 30 days. Besides, analyses were repeated using Fine and Grey's competing risk model to account for the competing risk of all-cause death.

We further assessed whether the relationship of pneumonia hospitalisation to the CVD risk was consistent across subpopulations: age at index date (<60 or ≥60 years), sex (men or women), region (rural or urban), educational attainment (no formal or primary school, or middle school or higher), smoking (current or noncurrent smoker), alcohol drinking (excessive or non-excessive drinker), BMI (<18.5, 18.5–27.9, or ≥28.0 kg/m^2^), waist circumference (male/female: <90/<85 or ≥90/≥85 cm), and family history of CVD (presence or absence). Subgroup analyses by disease status of hypertension, diabetes, and COPD (presence or absence) updated until the index date were also performed. The interactions between pneumonia hospitalisation and the abovementioned factors were tested using likelihood-ratio tests by comparing models with and without cross-product interaction terms.

To compare the relative importance of pneumonia hospitalisation with some traditional risk factors in terms of predicting the risk for incident CVD, we estimated the explained log-likelihood that was attributable to each factor over the 10-year follow-up (details in Supplementary Methods).^23^ We performed analyses using R version 4.0.3 and the packages “survival”, “Epi”, “cmprsk” and “rms”. All *P* values are 2-sided with a significance threshold at 0.05. Because of the potential for type I error owing to multiple comparisons, the findings for the analyses of five primary outcomes should be interpreted as substantially significant only when *P* < 0.01, based on the Bonferroni correction.

### Role of the funding source

The funders had no role in the study design, data collection, data analysis, data interpretation, or writing of the report. Y.H., Z.S., C.Y., and J.L. had access to dataset. All authors were responsible for the decision to submit for publication.

## Results

### Characteristics of the study population and the incidence of outcomes

There were 482,017 eligible CKB participants. A total of 24,060 had been hospitalised with pneumonia during follow-up, and 223,875 controls were obtained after EDS matching (Fig. 1). Among controls, 9159 (4.1%) were first drawn as controls and subsequently hospitalised with pneumonia. Of the 247,935 cases and controls that were included in the nested analysis cohort, the mean age (standard deviation) at baseline was 53.5 (10.4), 40.8% were men, and 61.8% resided in rural areas (Table 1). Despite matching by baseline age groups and decile of the frailty index, pneumonia cases had slightly higher age and frailty status than controls. Besides, compared to matched controls, pneumonia cases had worse self-reported health status and a higher prevalence of underlying conditions except for hypertension. Of the 23,686 participants who participated in both the baseline survey and the resurvey, most participants did not have their risk levels of lifestyle factors and medication use changed appreciably (Table S1). Changes in a small part of participants did not differ by disease status.

**Table 1:** Characteristics of the nested analysis cohorts (n = 247,935).

	Overall	Pneumonia cases (n = 24,060)	Matched controls (n = 223,875)	*P*-value
Sociodemographic characteristics				
Age at baseline, year (SD)	53.5 (10.4)	55.3 (10.5)	53.3 (10.4)	<0.0001
Age at index date, year (SD)	60.2 (10.2)	62.8 (10.6)	59.9 (10.1)	<0.0001
Men	101,159 (40.8)	10,118 (41.1)	91,041 (40.8)	0.35
Rural residence	153,281 (61.8)	15,212 (64.3)	138,069 (61.6)	<0.0001
Married	222,712 (89.8)	21,329 (90.7)	201,383 (89.7)	<0.0001
Middle school or higher	104,005 (41.9)	8823 (41.6)	95,182 (42.0)	0.23
Lifestyle factors				
Male current smokers^a^	68,425 (67.6)	7177 (71.0)	61,248 (67.3)	<0.0001
Female current smokers	4701 (3.2)	739 (3.2)	3962 (3.2)	0.62
Male excessive alcohol drinkers^b^	26,062 (25.8)	3102 (26.7)	22,960 (25.7)	0.02
Female excessive alcohol drinkers	2246 (1.5)	338 (1.5)	1908 (1.5)	0.32
Physical activity, MET h/d (SD)	20.8 (13.8)	21.0 (13.0)	20.8 (13.9)	0.0040
Central obesity^c^	59,309 (23.9)	5396 (23.0)	53,913 (24.0)	0.0006
BMI, kg/m^2^				<0.0001
<18.5	12,618 (5.1)	1800 (6.7)	10,818 (4.9)	
18.5–27.9	209,579 (84.5)	20,079 (83.6)	189,500 (84.7)	
≥28.0	25,738 (10.4)	2181 (9.8)	23,557 (10.4)	
Dietary habits				
Daily consumption of vegetables	234,950 (94.8)	23,194 (95.2)	211,756 (94.7)	0.01
Daily consumption of fresh fruits	39,958 (16.1)	3528 (15.4)	36,430 (16.2)	0.0005
Eating meat 1–6 days per week	137,954 (55.6)	14,558 (56.3)	123,396 (55.6)	0.02
Daily consumption of eggs	31,973 (12.9)	3007 (12.9)	28,966 (12.9)	0.84
Health status and medication				
Frailty	6263 (2.5)	897 (3.1)	5366 (2.4)	<0.0001
Self-reported poor health	25,768 (10.4)	3361 (12.6)	22,407 (10.1)	<0.0001
Using antihypertensive drugs	28,868 (11.6)	2710 (10.4)	26,158 (11.8)	<0.0001
Using glucose-lowering drugs	4575 (1.8)	529 (2.0)	4046 (1.8)	0.02
Medical history updated until index date				
Hypertension	93,975 (37.9)	8956 (35.1)	85,019 (38.2)	<0.0001
Diabetes	18,572 (7.5)	2254 (8.7)	16,318 (7.3)	<0.0001
COPD	24,751 (10.0)	4786 (15.7)	19,965 (9.2)	<0.0001
Tuberculosis	4718 (1.9)	876 (3.3)	3842 (1.7)	<0.0001
Asthma	2210 (0.9)	497 (2.0)	1713 (0.8)	<0.0001
Chronic kidney disease	3967 (1.6)	567 (2.0)	3400 (1.6)	<0.0001
Cirrhosis/chronic hepatitis	4080 (1.6)	660 (2.6)	3420 (1.5)	<0.0001
Peptic ulcer	11,509 (4.6)	1402 (5.5)	10,107 (4.5)	<0.0001
Gallstone diseases	20,281 (8.2)	2557 (9.6)	17,724 (8.0)	<0.0001
Rheumatoid arthritis	6028 (2.4)	749 (2.8)	5279 (2.4)	<0.0001
Fracture	22,672 (9.1)	2506 (9.9)	20,166 (9.1)	<0.0001
Cancer	6116 (2.5)	1494 (5.8)	4622 (2.1)	<0.0001
CVD family history at baseline^d^	48,732 (19.7)	4411 (19.3)	44,321 (19.7)	0.21

BMI, body mass index; COPD: chronic obstructive pulmonary disease; CVD, cardiovascular diseases; MET: metabolic equivalent of task; SD: standard deviation.

Data are shown as n (%) or mean (SD), and all variables were measured at baseline unless stated otherwise. Comparisons between groups were made using linear regression for continuous variables and logistic regression for categorical variables, with adjustments for age, sex, and study areas, as appropriate.

a Participants who quit smoking because of illness were also classified as current daily smokers.

b Excessive drinkers were those who drank ≥30 g of pure alcohol per day, or having stopped drinking.

c Central obesity was defined as waist circumference 90 cm or greater for men and 85 cm or greater for women.

d A CVD family history was defined as having at least one first-degree relative (biological father, mother, and siblings) with heart disease or stroke.

The mean time from baseline to the first pneumonia hospitalisation was 8.0 years, and that from matching to incident CVD ranged from 4.6–4.7 years. During long-term follow-up, 2389 (9.9%) pneumonia cases developed IHD (including 393 MCE and 1996 IHD other than MCE), 489 (2.0%) cases developed arrhythmia, 545 (2.3%) cases developed HF, 1764 (7.3%) cases developed IS (including 302 LACI and 1462 non-LACI), and 348 (1.4%) cases developed HS. The incidence (per 1000 person-years) of the five primary outcomes among pneumonia cases during 10-year follow-up was 32.4, 6.2, 7.3, 23.6, and 4.5, respectively. The corresponding figures for matched controls were 13.0, 2.0, 1.5, 14.6, and 3.1.

### Pneumonia hospitalisation with heart disease

**Fig. 2:**
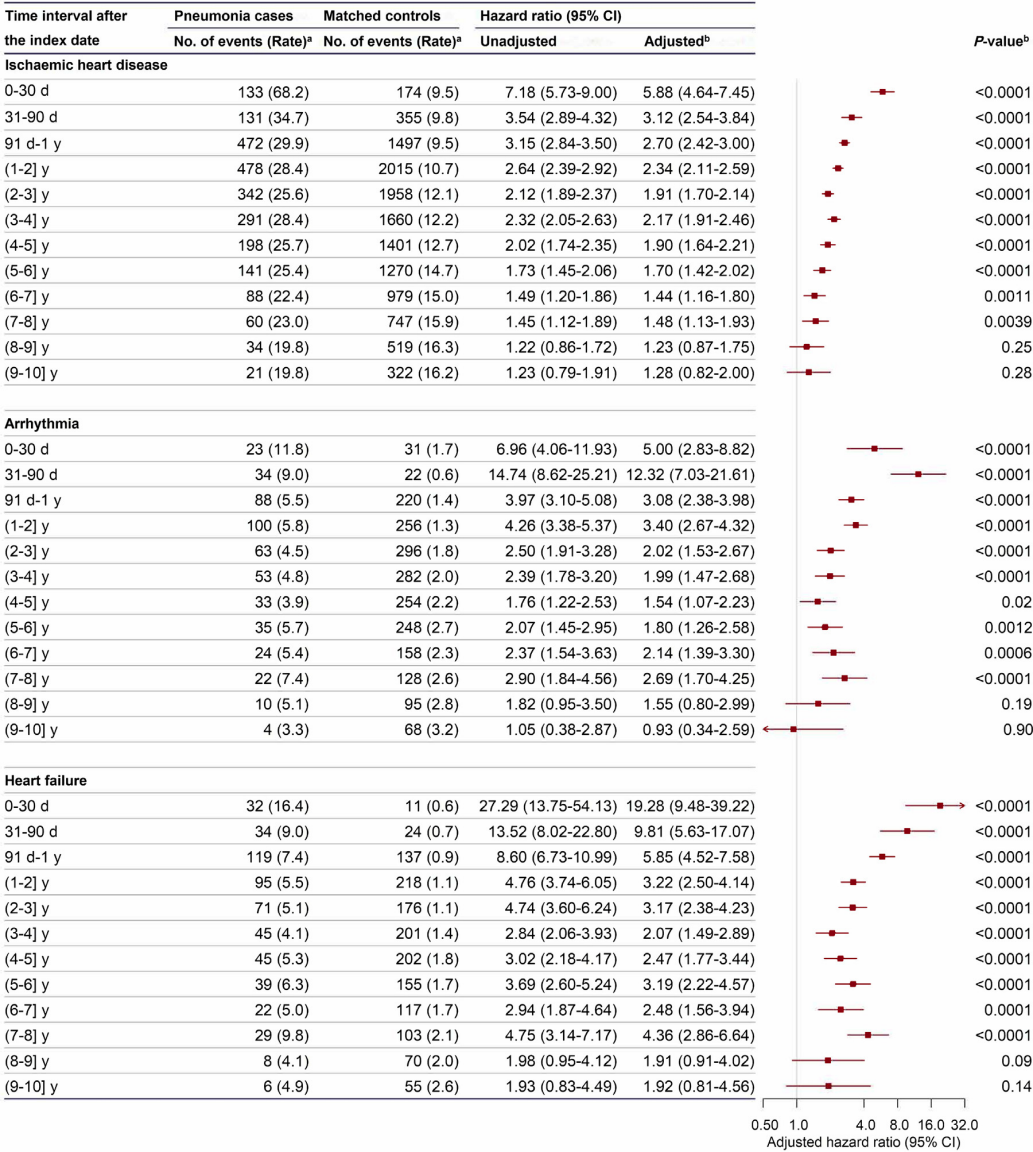
**Risk of ischaemic heart disease, arrhythmia, and heart failure after hospitalisation for pneumonia**. CI, confidence interval. The area of each square is inversely proportional to the variance, and 95% confidence intervals are shown. ^a^Rates are equal to the number of events per 1000 person-years. ^b^Multivariable models were stratified by age at index date in 5-year interval and study areas, and adjusted for sex, marital status, education, tobacco smoking, alcohol consumption, level of total physical activity, body mass index, waist circumference, intake frequency of vegetables, fresh fruits, eggs, and red meat, frailty index, self-reported health status, usage of antihypertensive or glucose-lowering drugs, major underlying conditions updated until the index date, and family history of cardiovascular disease. *P* values pertain to adjusted hazard ratios.

Compared to controls, pneumonia cases showed significantly increased incidences of IHD and its subtypes, arrhythmia, and HF from 1 month to several years after pneumonia hospitalisation (Fig. 2 and Fig. S1). The respective incidence (per 1000 person-years) of IHD, MCE, and IHD other than MCE, arrhythmia, and HF of pneumonia cases for the first 30 days were 68.2, 19.5, 48.7, 11.8, and 16.4. After adjustment for potential confounders, pneumonia cases had the highest risk of IHD for the first 30 days compared to matched controls, with an HR of 5.88 (95% CI: 4.64–7.45); and the HRs (95% CIs) for days 31–90 and day 91 to one year were 3.12 (2.54–3.84) and 2.70 (2.42–3.00), respectively (Fig. 2). The risk gradually decreased afterward but remained elevated until the eighth year, with an HR (95% CI) of 1.48 (1.13–1.93). The association patterns of pneumonia hospitalisation with MCE and IHD other than MCE were similar to IHD (Fig. S1).

Compared to matched controls, the risk of developing arrhythmia and HF increased markedly after pneumonia hospitalisation, with an HR (95% CI) of 5.00 (2.83–8.82) and 19.28 (9.48–39.22) for the first 30 days, respectively (Fig. 2). The risk gradually reduced afterward but remained significantly higher than controls until the eighth year for both arrhythmia and HF, with an HR (95% CI) of 2.69 (1.70–4.25) and 4.36 (2.86–6.64), respectively.

### Pneumonia hospitalisation and stroke

**Fig. 3:**
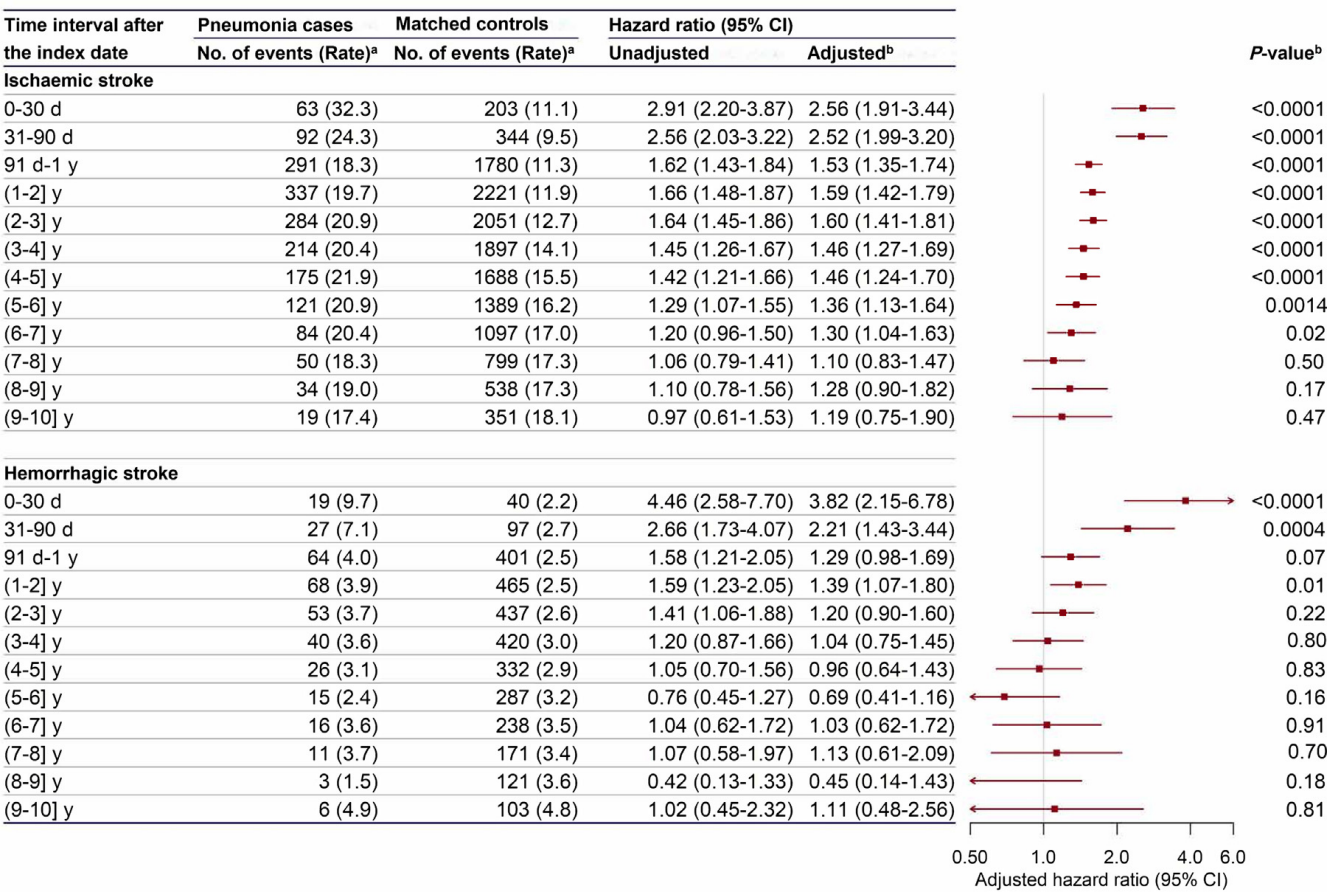
**Risk of ischaemic stroke and hemorrhagic stroke after hospitalisation for pneumonia.** CI, confidence interval. The area of each square is inversely proportional to the variance, and 95% confidence intervals are shown. ^a^Rates are equal to the number of events per 1000 person-years. ^b^Please refer to Fig. 2 for covariates adjusted in the models. *P* values pertain to adjusted hazard ratios.

The stroke incidence also increased after pneumonia hospitalisation (Fig. 3 and Fig. S2). The respective incidence (per 1000 person-years) of IS, LACI, non-LACI, and HS for the first 30 days after pneumonia hospitalisation were 32.3, 4.1, 28.2 and 9.7. Compared to matched controls, the risk of developing IS for the days 0–30 and days 31–90 after pneumonia hospitalisation was 2.56 (95% CI: 1.91–3.44) and 2.52 (1.99–3.20) for pneumonia cases, respectively (Fig. 3). The risk decreased afterward, with an HR (95% CI) of 1.53 (1.35–1.74) for day 91 to one year, and remained elevated until the seventh year after pneumonia hospitalisation (HR = 1.30; 95% CI: 1.04–1.63). The association patterns of LACI and non-LACI were similar to IS, though with wider CIs (Fig. S2).

The elevated risk of HS within the first 30 days after pneumonia hospitalisation was similar to IS, with an HR (95% CI) of 3.82 (2.15–6.78); the risk decreased afterward, but remained elevated during the second year after the index date, with an HR (95% CI) of 1.39 (1.07–1.80) (Fig. 3). After that, it did not differ between pneumonia cases and matched controls.

### Sensitivity analyses

The associations of pneumonia hospitalisation with incident CVD over 10 years were generally robust to sensitivity analyses (Tables S2–S6). Restricting pneumonia cases to those who were hospitalised with pneumonia in the first five years of follow-up led to a moderate reduction in the HRs and wider CIs, but the association pattern remained unchanged (Table S4). The competing risks model produced similar results to those in the primary analysis, indicating that the competing risk of all-cause mortality should have little influence on our results (Table S6).

### Subgroup analyses

The increased risk for five CVD outcomes following pneumonia was seen across all subgroups by sociodemographic factors, underlying conditions, lifestyle factors, and family history of CVD (Tables S7–S11). Despite having a lower incidence of HF than participants aged ≥60 years, those aged <60 years showed a higher relative risk of HF after pneumonia hospitalisation (*P*_*interaction*_ = 0.0020) (Table S9).

### The relative importance of pneumonia hospitalisation and traditional CVD risk factors

**Fig. 4:**
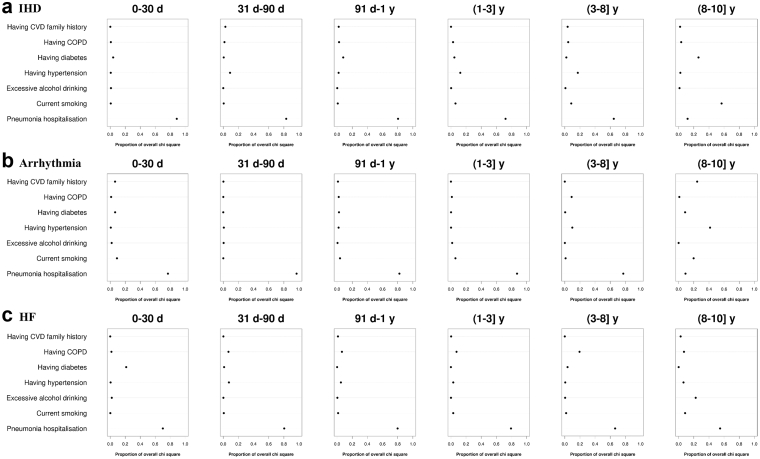
**Relative importance of pneumonia hospitalisation and traditional risk factors for CVD on the risk of ischaemic heart disease, arrhythmia, and heart failure during the 10-year follow-up**. CVD, cardiovascular disease; IHD, ischaemic heart disease; HF, heart failure; COPD, chronic obstructive pulmonary disease. Predictors were modeled as binary variables in Cox models. The relative importance of each predictor was measured by estimating explained log-likelihood, with a larger proportion of overall chi-square (higher value on the x-axis) indicating greater importance. Please refer to Supplementary Methods for the definition of predictors and covariates adjusted in the models.

**Fig. 5:**
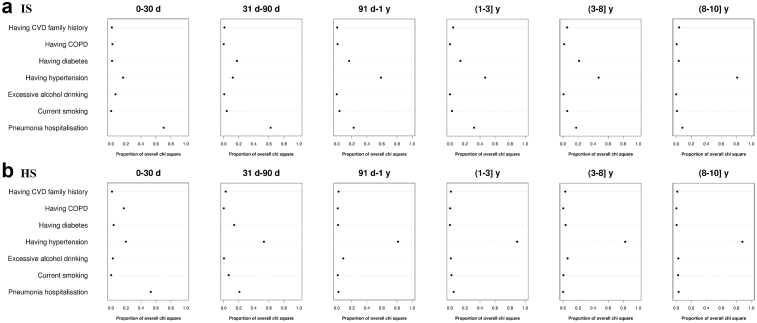
**Relative importance of pneumonia hospitalisation and traditional risk factors for CVD on the risk of ischaemic stroke and hemorrhagic stroke during the 10-year follow-up**. CVD, cardiovascular disease; IS, ischaemic stroke; HS, hemorrhagic stroke; COPD, chronic obstructive pulmonary disease. Predictors were modeled as binary variables in Cox models. The relative importance of each predictor was measured by estimating explained log-likelihood, with a larger proportion of overall chi-square (higher value on the x-axis) indicating greater importance. Please refer to Supplementary Methods for the definition of predictors and covariates adjusted in the models.

In terms of predicting the risk of heart disease, pneumonia hospitalisation was the strongest predictor until years 8–10 after the index date for HF, or until the 3–8 years for IHD and arrhythmia, and its relative importance was comparable to most traditional risk factors afterward (Fig. 4). As for the risk of IS and HS, pneumonia hospitalisation was one of the most important predictors for the first 90 days, and its relative importance in predicting IS and HS was close to most traditional risk factors afterward (Fig. 5).

## Discussion

In this large, matched cohort study nested within the CKB, pneumonia hospitalisation was associated with an increased risk of five CVD outcomes. The elevated risk was highest within the first 30 days after pneumonia hospitalisation, with subsequent risk reductions varying by CVD subtypes. The association estimates were highest for HF, with the elevated risk persisting for up to 8 years; followed by the risk of IHD, arrhythmia and IS, with the elevated risk persisting for around 7–8 years. The elevated risk of HS was mainly seen within the first two years. The above associations were consistently observed across various characteristics of the participants. In terms of relative importance in predicting the CVD risk compared with traditional CVD risk factors, pneumonia hospitalisation was the strongest predictor until years 8–10 after pneumonia hospitalisation for HF, and years 3–8 for IHD and arrhythmia, while predominantly in the first 90 days for IS and HS. Afterward, the relative importance of pneumonia hospitalisation decreased gradually and was comparable to most traditional risk factors.

Two matched cohort studies were conducted based on the Cardiovascular Health Study (CHS, age at enrollment ≥65 years) and the Atherosclerosis Risk in Communities study (ARIC, 45–64 years), including 591 and 680 pneumonia cases and matched controls followed for CVD occurrence over 10 years, respectively.^8^ The elevated risk of incident CVD, a composite endpoint of myocardial infarction, stroke, and fatal coronary heart disease, after pneumonia hospitalisation, remained up to the tenth year in CHS (HR = 1.86; 95% CI: 1.18–2.55) and the second year in ARIC (1.88; 1.10–2.66). Another Swedish cohort study of 236,739 men reported that hospital admission for sepsis or pneumonia was associated with an increased risk of IHD and stroke.^11^ The HR (95% CI) for 5+ years after hospitalisation was 1.64 (1.45–1.86) for IHD and 1.58 (1.26–1.98) for stroke. However, this study lacked data on potential confounders like smoking and underlying conditions, leaving possible confounding uncontrolled.

Two prospective cohort studies have consistently found that pneumonia was associated with an increased risk of HF. The CHS study of 5613 adults aged ≥65 years, with a mean follow-up of 11.9 years, showed a gradual decrease in the elevated HF risk after pneumonia hospitalisation but remained at the time interval of 5+ years (HR = 2.0; 95% CI: 1.56–2.58).^9^ A Canadian cohort study included 4988 pneumonia cases and 23,060 matched controls, with a median follow-up of 9.9 years.^10^ The average age of pneumonia cases was 55 years, and 63.4% were managed as outpatients. The HF risk was higher in pneumonia cases than in controls, with an HR of 1.61 (95% CI: 1.44–1.81). Besides, the association estimate of HF risk associated with pneumonia was higher in inpatients (1.94; 1.64–2.29) than in outpatients (1.33; 1.12–1.57).

Our findings are consistent with the above studies and found that pneumonia hospitalisation was associated with long-term cardiovascular risk, including increased risk of IHD, HF, and IS, in the Chinese population. We further extend the evidence that the risk of arrhythmia and HS also increased after pneumonia hospitalisation. Differences existed in the strength of association and duration of elevated risk among five CVD subtypes, with the strongest association and longest duration up to 8 years for the risk of heart disease after pneumonia hospitalisation, and a relatively weaker association and shortest duration for HS risk. The mechanisms that underlie the associations remain not fully understood and may be related to the local or systemic inflammatory response, biomechanical stress and vasoconstriction, endothelial damage, cardiac autonomic dysfunction, and prothrombotic and procoagulant states after pneumonia episodes.^3^^,^^24^, ^25^, ^26^, ^27^ The pathophysiological changes can persist after clinically recovered from pneumonia,^5^^,^^6^ lending support for the association.

The present study showed that the increased risk of five CVD outcomes following pneumonia was seen across all subgroups. Although adults aged ≥60 years had a higher incidence of HF, the association estimates of developing HF associated with pneumonia hospitalisation was higher in adults aged <60 years. The potential explanation for this result is that pneumonia adds a relatively small additional risk for older adults who already have a high disease risk. The above Canadian cohort study has also got similar findings for age differences.^10^

The present study is the first large matched cohort study that examined the associations of pneumonia hospitalisation with the long-term risk of five CVD subtypes using the same analysis strategy. The long follow-up period and large sample size ensured statistical power to detect the changes in the strength of association in different periods over 10 years after pneumonia. In the study of pneumonia and CVD, some potential confounding factors, including age, frailty status, underlying conditions, and lifestyle, must be considered and controlled. The collection of detailed baseline information and comprehensive linkage to established registries and HI database allowed us to address confounding bias from these factors by either matching or adjusting in the models. In addition, our study includes a nationwide geographically spread population characterized by a wide range of ages, socioeconomic characteristics, and health status, thus strengthening the external validity of our results.

Our study also has some limitations. First, we did not include mildly ill patients with pneumonia who were treated in outpatient settings. Prior studies found an increased risk of CVD even in outpatients with pneumonia.^10^ However, as the severity of pneumonia increased, the association between pneumonia and subsequent CVD risk was stronger and lasted longer.^8^ We also did not collect information on the severity of pneumonia. Length of hospital stay of pneumonia was not found to be a good indicator of severity, since it may be influenced by HI policies in China.^28^ Besides, patients with a poor prognosis might be discharged home in a terminal stage,^29^ resulting in a shorter hospital stay. Second, we could not distinguish community-acquired pneumonia from hospital-acquired pneumonia according to information from HI data. However, our findings were generally unchanged when restricted the exposed group to participants without any other hospital admission in the previous 30 days. Third, we did not collect information on vaccination. However, pneumonia and influenza vaccination rates in middle-aged and older adults in China are very low (<5%),^30^ and therefore unlikely to influence our findings. Fourth, we did not have information on the causative pathogen of pneumonia. The identification of pneumonia pathogens remains difficult in routine clinical practice.^2^ Prior evidence has shown that despite more sensitive and specific diagnostic methods being applied, pathogens were detected in only 38% of patients with pneumonia.^31^ Fifth, information on lifestyle and medication use was only collected at baseline. The potential effect of any changes in these factors over time on the associations of interest could not be ascertained.

In recent years, the long-term cardiovascular consequences of coronavirus disease 2019 (COVID-19) are a persistent public health concern.^32^ The results of the present large prospective cohort study of Chinese population suggest that pneumonia caused by pathogens other than severe acute respiratory syndrome coronavirus 2 was associated with short- and long-term CVD risk, with an elevated risk of certain CVD outcomes persisted for up to 8 years. The ageing population and increasing prevalence of chronic diseases contribute to the substantial burden of pneumonia hospitalisation in middle-aged and older adults.^33^^,^^34^ Together with the pandemic of COVID-19, these factors, alone or in combination, would pose a great burden for the prevention and control of CVD in the near future.^35^ Therefore, prevention of all-cause pneumonia and particular attention to short- and long-term cardiovascular health of patients recovered from pneumonia are important ways of reducing the burden of CVD.

## Contributors

J.L. conceived and designed the paper. L.L., Z.C., and J.C., as the members of CKB steering committee, designed and supervised the conduct of the whole study, obtained funding, and together with C.Y., Y.G., P.P., L.Y., Y.C., H.D., X.T., S.G., D.A., Y.P. and D.S. acquired the data. Y.H., Z.S., C.Y., and J.L. have accessed and verified the dataset. Y.H. and Z.S. analysed the data. Y.H. drafted the manuscript. J.L. and L.L. contributed to the interpretation of the results. J.L. critically reviewed and revised the manuscript for important intellectual content. All authors reviewed and approved the final manuscript. J.L. is the guarantor.

## Data sharing statement

The access policy and procedures are available at www.ckbiobank.org.

## Declaration of interests

All authors declare no competing interests.

## Appendix A Supplementary data

Supplementary Methods, Tables S1–S11, Figures S1 and S2

Caption for Supplementary Material

List of All Study Group Members

Translated Abstract

## References

[1] GBD 2017 Causes of Death Collaborators (2018). Global, regional, and national age-sex-specific mortality for 282 causes of death in 195 countries and territories, 1980-2017: a systematic analysis for the Global Burden of Disease Study 2017. Lancet.

[2] Quinton L.J., Walkey A.J., Mizgerd J.P. (2018). Integrative physiology of pneumonia. Physiol Rev.

[3] Corrales-Medina V.F., Musher D.M., Shachkina S., Chirinos J.A. (2013). Acute pneumonia and the cardiovascular system. Lancet.

[4] Corrales-Medina V.F., Madjid M., Musher D.M. (2010). Role of acute infection in triggering acute coronary syndromes. Lancet Infect Dis.

[5] Yende S., D'Angelo G., Mayr F. (2011). Elevated hemostasis markers after pneumonia increases one-year risk of all-cause and cardiovascular deaths. PLoS One.

[6] Menendez R., Mendez R., Aldas I. (2019). Community-acquired pneumonia patients at risk for early and long-term cardiovascular events are identified by cardiac biomarkers. Chest.

[7] Hu Y., Yu C., Guo Y. (2021). Pneumonia hospitalizations and the subsequent risk of incident ischaemic cardiovascular disease in Chinese adults. Int J Epidemiol.

[8] Corrales-Medina V.F., Alvarez K.N., Weissfeld L.A. (2015). Association between hospitalization for pneumonia and subsequent risk of cardiovascular disease. JAMA.

[9] Corrales-Medina V.F., Taljaard M., Yende S. (2015). Intermediate and long-term risk of new-onset heart failure after hospitalization for pneumonia in elderly adults. Am Heart J.

[10] Eurich D.T., Marrie T.J., Minhas-Sandhu J.K., Majumdar S.R. (2017). Risk of heart failure after community acquired pneumonia: prospective controlled study with 10 years of follow-up. BMJ.

[11] Bergh C., Fall K., Udumyan R., Sjoqvist H., Frobert O., Montgomery S. (2017). Severe infections and subsequent delayed cardiovascular disease. Eur J Prev Cardiol.

[12] Corrales-Medina V.F., Suh K.N., Rose G. (2011). Cardiac complications in patients with community-acquired pneumonia: a systematic review and meta-analysis of observational studies. PLoS Med.

[13] Chen Z., Lee L., Chen J. (2005). Cohort profile: the Kadoorie Study of Chronic Disease in China (KSCDC). Int J Epidemiol.

[14] Chen Z., Chen J., Collins R. (2011). China Kadoorie Biobank of 0.5 million people: survey methods, baseline characteristics and long-term follow-up. Int J Epidemiol.

[15] Yang G., Hu J., Rao K.Q., Ma J., Rao C., Lopez A.D. (2005). Mortality registration and surveillance in China: history, current situation and challenges. Popul Health Metrics.

[16] Ohneberg K., Beyersmann J., Schumacher M. (2019). Exposure density sampling: dynamic matching with respect to a time-dependent exposure. Stat Med.

[17] Ohneberg K. (2019).

[18] Fan J., Yu C., Guo Y. (2020). Frailty index and all-cause and cause-specific mortality in Chinese adults: a prospective cohort study. Lancet Public Health.

[19] Lv J., Qi L., Yu C. (2015). Consumption of spicy foods and total and cause specific mortality: population based cohort study. BMJ.

[20] Li J., Qin C., Lv J. (2019). Solid fuel use and incident COPD in Chinese adults: findings from the China Kadoorie Biobank. Environ Health Perspect.

[21] Dekker F.W., de Mutsert R., van Dijk P.C., Zoccali C., Jager K.J. (2008). Survival analysis: time-dependent effects and time-varying risk factors. Kidney Int.

[22] Sergi G., Veronese N., Fontana L. (2015). Pre-frailty and risk of cardiovascular disease in elderly men and women: the Pro.V.A. study. J Am Coll Cardiol.

[23] Rawshani A., Rawshani A., Franzen S. (2018). Risk factors, mortality, and cardiovascular outcomes in patients with type 2 diabetes. N Engl J Med.

[24] Singanayagam A., Singanayagam A., Elder D.H., Chalmers J.D. (2012). Is community-acquired pneumonia an independent risk factor for cardiovascular disease?. Eur Respir J.

[25] Musher D.M., Abers M.S., Corrales-Medina V.F. (2019). Acute infection and myocardial infarction. N Engl J Med.

[26] Groenewegen A., Rutten F.H., Mosterd A., Hoes A.W. (2020). Epidemiology of heart failure. Eur J Heart Fail.

[27] Restrepo M.I., Reyes L.F. (2018). Pneumonia as a cardiovascular disease. Respirology.

[28] Gao C., Xu F., Liu G.G. (2014). Payment reform and changes in health care in China. Soc Sci Med.

[29] Weng L., Hu Y., Sun Z. (2022). Place of death and phenomenon of going home to die in Chinese adults: a prospective cohort study. Lancet Reg Health West Pac.

[30] Fan J., Cong S., Wang N. (2020). Influenza vaccination rate and its association with chronic diseases in China: results of a national cross-sectional study. Vaccine.

[31] Jain S., Self W.H., Wunderink R.G. (2015). Community-acquired pneumonia requiring hospitalization among U.S. adults. N Engl J Med.

[32] Xie Y., Xu E., Bowe B., Al-Aly Z. (2022). Long-term cardiovascular outcomes of COVID-19. Nat Med.

[33] Hu Y., Han Y., Yu C. (2022). The hospitalization burden of all-cause pneumonia in China: a population-based study, 2009-2017. Lancet Reg Health West Pac.

[34] Smith S.B., Ruhnke G.W., Weiss C.H., Waterer G.W., Wunderink R.G. (2014). Trends in pathogens among patients hospitalized for pneumonia from 1993 to 2011. JAMA Intern Med.

[35] Driggin E., Madhavan M.V., Bikdeli B. (2020). Cardiovascular considerations for patients, health care workers, and health systems during the COVID-19 pandemic. J Am Coll Cardiol.

