# Supplementary material for: Association between pneumonia hospitalisation and long-term risk of cardiovascular disease in Chinese adults: A prospective cohort study
Source: eClinicalMedicine. 2022 Dec 2;55:101761. doi: 10.1016/j.eclinm.2022.101761 (PMC9722470; doi:10.1016/j.eclinm.2022.101761)
Supplement: Supplementary Methods, Tables S1–S11, Figures S1 and S2 [file mmc1.pdf]

Table of Contents

Page 2: Members of the China Kadoorie Biobank collaborative group

Page 3: Supplementary Methods

Page 5: Table S1. Changes in lifestyle factors and medication between 2004-08 baseline and 2013-14 resurvey by disease status at 2013-14 resurvey in 23,686 participants

Page 7: Table S2. Sensitivity analyses for association of pneumonia hospitalisation with new-onset cardiovascular disease by excluding participants who had multiple pneumonia hospitalisations before incident cardiovascular disease

Page 8: Table S3. Sensitivity analyses for association of pneumonia hospitalisation with new-onset cardiovascular disease by restricting the primary analysis to participants who were categorized as robust according to frailty index at baseline

Page 9: Table S4. Sensitivity analyses for association of pneumonia hospitalisation with new-onset cardiovascular disease by restricting the exposed group to participants hospitalised for pneumonia during the first five years of follow-up

Page 10: Table S5. Sensitivity analyses for association of pneumonia hospitalisation with new-onset cardiovascular disease by restricting the exposed group to participants without any other hospital admission in the previous 30 days

Page 11: Table S6. Sensitivity analyses for association of pneumonia hospitalisation with new-onset cardiovascular disease using competing risk model to account for the competing risk of death

Page 12: Table S7. Adjusted hazard ratios (95% CIs) for incident ischaemic heart disease by subgroups after hospitalisation for pneumonia

Page 15: Table S8. Adjusted hazard ratios (95% CIs) for incident arrhythmia by subgroups after hospitalisation for pneumonia

Page 18: Table S9. Adjusted hazard ratios (95% CIs) for incident heart failure by subgroups after hospitalisation for pneumonia

Page 21: Table S10. Adjusted hazard ratios (95% CIs) for incident ischaemic stroke by subgroups after hospitalisation for pneumonia

Page 24: Table S11. Adjusted hazard ratios (95% CIs) for incident hemorrhagic stroke by subgroups after hospitalisation for pneumonia

Page 27: Fig. S1. Risk of ischaemic heart disease by subtypes after hospitalisation for pneumonia

Page 28: Fig. S2. Risk of ischaemic stroke by subtypes after hospitalisation for pneumonia

Members of the China Kadoorie Biobank collaborative group

International Steering Committee: Junshi Chen, Zhengming Chen (PI), Robert Clarke, Rory Collins, Yu Guo, Liming Li (PI), Jun Lv, Richard Peto, Robin Walters.
International Co-ordinating Centre, Oxford: Daniel Avery, Derrick Bennett, Ruth Boxall, Sue Burgess, Ka Hung Chan, Yumei Chang, Yiping Chen, Zhengming Chen, Johnathan Clarke; Robert Clarke, Huaidong Du, Ahmed Edris Mohamed, Zammy Fairhurst-Hunter, Hannah Fry, Simon Gilbert, Alex Hacker, Mike Hill, Michael Holmes, Pek Kei Im, Andri Iona, Maria Kakkoura, Christiana Kartsonaki, Rene Kerosi, Kuang Lin, Mohsen Mazidi, Iona Millwood, Sam Morris, Qunhua Nie, Alfred Pozarickij, Paul Ryder, Saredo Said, Sam Sansome, Dan Schmidt, Paul Sherliker, Rajani Sohoni, Becky Stevens, Iain Turnbull, Robin Walters, Lin Wang, Neil Wright, Ling Yang, Xiaoming Yang, Pang Yao.
National Co-ordinating Centre, Beijing: Yu Guo, Xiao Han, Can Hou, Jun Lv, Pei Pei, Chao Liu, Canqing Yu, Qingmei Xia. **10 Regional Co-ordinating Centres: Qingdao CDC:** Zengchang Pang, Ruqin Gao, Shanpeng Li, Haiping Duan, Shaojie Wang, Yongmei Liu, Ranran Du, Yajing Zang, Liang Cheng, Xiaocao Tian, Hua Zhang, Yaoming Zhai, Feng Ning, Xiaohui Sun, Feifei Li. **Licang CDC:** Silu Lv, Junzheng Wang, Wei Hou. **Heilongjiang Provincial CDC:** Wei Sun, Shichun Yan, Xiaoming Cui. **Nangang CDC:** Chi Wang, Zhenyuan Wu,Yanjie Li, Quan Kang. **Hainan Provincial CDC:** Huiming Luo, Tingting Ou. **Meilan CDC:** Xiangyang Zheng, Zhendong Guo, Shukuan Wu, Yilei Li, Huimei Li. **Jiangsu Provincial CDC:** Ming Wu, Yonglin Zhou, Jinyi Zhou, Ran Tao, Jie Yang, Jian Su. **Suzhou CDC:** Fang Liu, Jun Zhang, Yihe Hu, Yan Lu, Liangcai Ma, Aiyu Tang, Shuo Zhang, Jianrong Jin, Jingchao Liu. **Guangxi Provincial CDC:** Mei Lin, Zhenzhen Lu. **Liuzhou CDC:** Lifang Zhou, Changping Xie, Jian Lan, Tingping Zhu, Yun Liu, Liuping Wei, Liyuan Zhou, Ningyu Chen, Yulu Qin, Sisi Wang. **Sichuan Provincial CDC:** Xianping Wu, Ningmei Zhang, Xiaofang Chen, Xiaoyu Chang. **Pengzhou CDC:** Mingqiang Yuan, Xia Wu, Xiaofang Chen, Wei Jiang, Jiaqiu Liu, Qiang Sun. **Gansu Provincial CDC:** Faqing Chen, Xiaolan Ren, Caixia Dong. **Maiji CDC:** Hui Zhang, Enke Mao, Xiaoping Wang, Tao Wang, Xi zhang. **Henan Provincial CDC:** Kai Kang, Shixian Feng, Huizi Tian, Lei Fan. **Huixian CDC:** XiaoLin Li, Huarong Sun, Pan He, Xukui Zhang. **Zhejiang Provincial CDC:** Min Yu, Ruying Hu, Hao Wang. **Tongxiang CDC**: Xiaoyi Zhang, Yuan Cao, Kaixu Xie, Lingli Chen, Dun Shen. **Hunan Provincial CDC:** Xiaojun Li, Donghui Jin, Li Yin, Huilin Liu, Zhongxi Fu. **Liuyang CDC:** Xin Xu, Hao Zhang, Jianwei Chen,Yuan Peng, Libo Zhang, Chan Qu.

Supplementary Methods

The ongoing outcome adjudication process of cardiovascular events in the China Kadoorie Biobank (CKB) cohort

The ongoing outcome adjudication process of cardiovascular events in the CKB cohort has been initiated since 2014. The medical records of incident cases were retrieved, and the diagnosis was confirmed by cardiovascular specialists blinded to the study assay using a standardized verification process. By October 2018, of 33,515 incident ischaemic heart disease cases and 40,465 stroke cases reported since the baseline survey whose medical records have been retrieved, 88% of ischaemic heart disease and 92% of stroke cases were confirmed for diagnosis, indicating a relatively high diagnostic accuracy.

Detailed information on the selection of the analysis cohort

The exposure density sampling is an efficient risk set sampling to perform dynamic matching for a time-dependent exposure occurring over time. Considering that the occurrence of a cardiovascular event may affect the incidence of the subsequent outcomes (e.g., individuals with arrhythmia were also more likely to develop heart failure), we used a composite endpoint of incident cardiovascular disease, including ischaemic heart disease, arrhythmia, heart failure, ischaemic stroke, and hemorrhagic stroke, for sampling. Individuals were removed from the risk sets at the time of pneumonia hospitalisation, diagnosis of cardiovascular disease outcomes, death, loss to follow-up, or December 31, 2018, whichever occurred first. Briefly, a total of 24,060 participants hospitalised for pneumonia were included in the following matching process. For each exposed individual, up to 15 matched controls who were still at risk for incident cardiovascular disease and still non-exposed at the time at which the exposed one was hospitalised for pneumonia (i.e., the index date), were selected from the risk set. Besides matching for time to exposure, other matched factors included baseline age groups with 5-year increments, sex, urban or rural area, and decile of the frailty index (Fan, Yu et al. 2020). The sampling of controls was carried out with replacement to derive unbiased estimators; therefore, a control can be sampled multiple times and serve as controls for more than one exposed individual (Ohneberg 2019, Ohneberg, Beyersmann et al. 2019). In this scenario, their time of the first sampling as a control will be considered the left-truncation entry time. A sampled control could also be hospitalised with pneumonia and become exposed later. For such controls, the first sampling time was the entry time, and the exposure was included as a time-dependent variable (Ohneberg 2019).

Covariates adjusted for in the multivariable models

The multivariable models were stratified by age at index date in the 5-year interval and study area, and adjusted for sex (men or women), marital status (married or others), education (no formal school, primary school, middle school, high school, college, or university or higher), tobacco smoking (noncurrent smokers, current smokers or past smokers who quit due to illness: 1-14, 15-24, or ≥25 cigarettes or equivalent per day), alcohol consumption (never weekly drinkers, former weekly drinkers, weekly but not daily drinkers, daily drinkers of <30 g , or ≥30 g of pure alcohol per day), level of total physical activity (metabolic equivalent of tasks [h/d]), body mass index (kg/m^2), waist circumference (mm), daily consumption of vegetables, fresh fruits, and eggs, and eating meat 1-6 days per week (yes or no), frailty index, self-reported health status (poor, not poor), usage of antihypertensive or glucose-

lowering drugs (yes or no), major underlying conditions updated until the index date (presence or absence), and family history of cardiovascular disease (yes or no).

Analysis of relative importance for various risk factors

Log likelihoods are useful for quantifying the predictive information contained in a predictor compared with the information contained in the entire set of predictors. In line with the previous studies (Rawshani et al. 2018), we chose to calculate the relative importance as measured by the proportion of explainable log-likelihood explained by pneumonia hospitalisation and other traditional risk factors for cardiovascular disease. To capture the relative importance of risk factors over 10-years, we constructed separate Cox models for each time interval for the 10-year follow-up for each primary outcome. The Cox models were stratified by age at index date in 5-year interval and study areas, and all predictors were modeled as binary variables. Participants who quit smoking because of illness were also classified as current smokers. Excessive alcohol drinking was defined as a consumption of ≥30 g of pure alcohol per day, or having stopped drinking. Other covariates included in the Cox models for this analysis and their form were consistent with the primary analysis. The partial effect of each risk factor was quantified by computing the proportion of explainable log-likelihood explained by each risk factor, i.e., the Wald χ^2 statistics minus the degree of freedom. If a predictor interacts with another predictor, the χ^2 combines the interaction with the main effects. We used the rms package (version 6.2.0) in R software for this analysis.

Table S1. Changes in lifestyle factors and medication between 2004-08 baseline and 2013-14 resurvey by disease status at 2013-14 resurvey in 23,686 participants

	Without pneumonia and CVD[a]	With either or both of pneumonia and CVD[a]	Total
**No. of participants, n**	21771	1915	23686
**Age at baseline, year (SD)**	50.0 (9.9)	56.5 (9.5)	50.5 (10.0)
**Age at resurvey, year (SD)**	58.0 (9.9)	64.5 (9.6)	58.5 (10.0)
**Physical activity, MET-h/d (SD)**	-3.3 (16.3)	-4.4 (13.9)	-3.3 (16.1)
**Smoking**			
Stable	93.3 (93.0-93.6)	91.9 (90.8-93.0)	93.1 (92.8-93.5)
Worse	1.5 (1.3-1.6)	1.3 (0.8-1.8)	1.4 (0.5-3.3)
Better	5.3 (5.0-5.5)	6.8 (5.8-7.8)	5.4 (4.2-6.8)
**Alcohol intake**			
Stable	90.8 (90.4-91.2)	89.3 (88.0-90.6)	90.7 (90.3-91.0)
Worse	5.1 (4.8-5.4)	5.8 (4.9-6.8)	5.2 (4.0-6.6)
Better	4.1 (3.8-4.3)	4.8 (3.9-5.8)	4.2 (3.0-5.6)
**Dietary habits**			
Stable	95.3 (95.0-95.6)	95.7 (94.9-96.5)	95.3 (95.0-95.6)
Worse	1.2 (1.1-1.4)	1.0 (0.6-1.4)	1.0 (0.2-3.0)
Better	3.5 (3.2-3.7)	3.3 (2.6-4.0)	3.4 (2.3-4.9)
**Body mass index**			
Stable	89.4 (89.0-89.8)	87.5 (86.1-89.0)	89.2 (88.8-89.6)
Worse	6.4 (6.1-6.8)	8.2 (7.0-9.4)	6.6 (5.4-8.0)
Better	4.2 (3.9-4.5)	4.3 (3.4-5.2)	4.2 (3.1-5.7)
**Waist circumference**			
Stable	76.1 (75.6-76.7)	75.1 (73.1-77.1)	76.1 (75.4-76.7)
Worse	21.4 (20.9-21.9)	22.3 (20.4-24.2)	21.5 (20.3-22.6)
Better	2.5 (2.3-2.7)	2.6 (2.0-3.2)	2.6 (1.4-4.2)
**Usage of antihypertensive drugs**			
Remained unchanged	84.5 (84.0-85.0)	71.9 (69.9-73.8)	83.4 (82.8-83.9)
Cessation of medication	1.2 (1.1-1.4)	1.5 (1.0-2.0)	1.3 (0.4-3.4)
Medication initiation	14.3 (13.8-14.7)	26.6 (24.7-28.6)	15.4 (14.2-16.6)
**Usage of glucose-lowering drugs**			
Remained unchanged	96.8 (96.6-97.1)	95.6 (94.7-96.5)	96.7 (96.5-96.9)
Cessation of medication	0.4 (0.3-0.5)	0.4 (0.2-0.6)	0.0 (0.0-3.8)
Medication initiation	2.8 (2.6-3.0)	4.0 (3.1-4.8)	2.9 (1.8-4.5)

CVD, cardiovascular disease; SD: standard deviation; MET-h/d, metabolic equivalent of task hours per day.

[a] A composite endpoint of incident cardiovascular disease, including arrhythmia, ischaemic heart disease, heart failure, ischaemic stroke, and hemorrhagic stroke.

All variables were adjusted for age at baseline, sex, and study areas, as appropriate. Data were presented as percentages (95% confidence intervals) unless otherwise indicated. To facilitate presentation, we grouped lifestyle factors into high-risk and low-risk groups based on our previous study (Han, Hu et al. 2021). The definition of high-risk lifestyle factors were as follows: current smoking or having stopped because of illness; daily drinking ≥30g of pure alcohol or having stopped drinking habit; non-daily eating of vegetables, fruits, and eggs, and eating red meat daily or less than weekly; having BMI <18.5 or ≥28.0 kg/m^2; having waist circumference ≥90cm (men) / 85cm (women). For physical activity, we directly calculated the difference between baseline and resurvey. Changes in other lifestyle factors between baseline and resurvey were classified into stable (at the same risk level), better (from high-risk to low-risk lifestyle), and worse (from low-risk to high-risk lifestyle).

Table S2. Sensitivity analyses for association of pneumonia hospitalisation with new-onset cardiovascular disease by excluding participants who had multiple pneumonia hospitalisations before incident cardiovascular disease

Time interval after the index date	Adjusted hazard ratio (95% CI)		
	**Ischaemic heart disease**	**Arrhythmia**	**Heart failure**
**0-90 d**	4.58 (3.93-5.35)	6.04 (3.82-9.56)	13.45 (8.79-20.58)
**91 d-1 y**	2.71 (2.43-3.04)	2.52 (1.91-3.34)	5.24 (4.00-6.86)
**(1-2] y**	2.38 (2.13-2.66)	2.80 (2.16-3.63)	3.09 (2.30-4.16)
**(2-3] y**	2.09 (1.84-2.38)	1.68 (1.20-2.34)	2.53 (1.82-3.52)
**(3-4] y**	2.00 (1.74-2.31)	1.77 (1.25-2.50)	1.89 (1.28-2.79)
**(4-5] y**	1.81 (1.53-2.14)	1.40 (0.90-2.19)	2.12 (1.44-3.13)
**(5-6] y**	1.64 (1.33-2.00)	1.97 (1.32-2.93)	2.27 (1.47-3.52)
**(6-7] y**	1.49 (1.16-1.92)	2.03 (1.20-3.44)	2.58 (1.51-4.40)
**(7-8] y**	1.44 (1.06-1.96)	1.93 (1.12-3.31)	3.72 (2.32-5.98)
**(8-9] y**	1.04 (0.68-1.61)	1.41 (0.65-3.08)	2.46 (1.05-5.81)
**(9-10] y**	1.20 (0.72-2.01)	0.98 (0.30-3.18)	2.48 (0.84-7.29)
	**Ischaemic stroke**	**Hemorrhagic stroke**	
**0-90 d**	2.81 (2.34-3.38)	2.78 (1.77-4.36)	
**91 d-1 y**	1.72 (1.51-1.96)	1.44 (1.10-1.89)	
**(1-2] y**	1.75 (1.54-1.98)	1.39 (1.06-1.84)	
**(2-3] y**	1.57 (1.37-1.80)	1.33 (0.97-1.83)	
**(3-4] y**	1.48 (1.26-1.74)	1.27 (0.89-1.80)	
**(4-5] y**	1.53 (1.28-1.82)	0.92 (0.59-1.42)	
**(5-6] y**	1.37 (1.11-1.69)	0.66 (0.37-1.18)	
**(6-7] y**	1.36 (1.05-1.75)	1.22 (0.69-2.16)	
**(7-8] y**	1.16 (0.82-1.63)	0.96 (0.47-1.96)	
**(8-9] y**	1.23 (0.83-1.80)	0.31 (0.08-1.28)	
**(9-10] y**	1.24 (0.76-2.04)	1.42 (0.56-3.57)	

CI, confidence interval.

The first two period after the index date in the primary analysis were merged into days 0-90 due to small sample size. Multivariable models were stratified by age at index date in 5-year interval and study areas, and adjusted for sex, marital status, education, tobacco smoking, alcohol consumption, level of total physical activity, body mass index, waist circumference, intake frequency of vegetables, fresh fruits, eggs, and red meat, frailty index, self-reported health status, usage of antihypertensive or glucose-lowering drugs, major underlying conditions updated until the index date, and family history of cardiovascular disease.

Table S3. Sensitivity analyses for association of pneumonia hospitalisation with new-onset cardiovascular disease by restricting the primary analysis to participants who were categorized as robust according to frailty index at baseline

**Time interval after the index date**	**Adjusted hazard ratio (95% CI)**		
	**Ischaemic heart disease**	**Arrhythmia**	**Heart failure**
**0-30 d**	6.49 (4.42-9.54)	6.51 (2.91-14.56)	14.16 (4.71-42.52)
**31-90 d**	3.55 (2.54-4.95)	6.76 (3.19-14.34)	21.61 (7.57-61.71)
**91 d-1 y**	3.08 (2.57-3.69)	3.85 (2.56-5.78)	7.96 (4.97-12.74)
**(1-2] y**	2.55 (2.14-3.04)	2.95 (1.99-4.35)	4.03 (2.32-6.99)
**(2-3] y**	2.24 (1.85-2.72)	3.17 (2.10-4.77)	2.91 (1.69-5.02)
**(3-4] y**	2.24 (1.81-2.78)	2.04 (1.23-3.39)	2.21 (1.14-4.29)
**(4-5] y**	1.81 (1.40-2.35)	1.29 (0.67-2.48)	3.36 (1.83-6.17)
**(5-6] y**	1.62 (1.20-2.18)	1.22 (0.58-2.53)	1.84 (0.82-4.12)
**(6-7] y**	1.35 (0.94-1.92)	2.13 (1.15-3.94)	3.85 (2.10-7.06)
**(7-8] y**	1.56 (1.03-2.37)	2.94 (1.30-6.65)	2.62 (1.08-6.34)
**(8-9] y**	1.17 (0.61-2.23)	1.19 (0.36-3.89)	0.89 (0.12-6.83)
**(9-10] y**	1.80 (0.93-3.50)	2.37 (0.53-10.69)	5.16 (1.60-16.60)
	**Ischaemic stroke**	**Hemorrhagic stroke**	
**0-30 d**	2.54 (1.50-4.28)	4.71 (1.97-11.28)	
**31-90 d**	2.20 (1.43-3.40)	2.57 (1.26-5.26)	
**91 d-1 y**	1.77 (1.45-2.16)	1.35 (0.87-2.11)	
**(1-2] y**	1.86 (1.54-2.24)	1.34 (0.87-2.08)	
**(2-3] y**	1.58 (1.27-1.97)	0.94 (0.55-1.63)	
**(3-4] y**	1.50 (1.18-1.91)	0.91 (0.47-1.76)	
**(4-5] y**	1.64 (1.27-2.12)	1.51 (0.82-2.76)	
**(5-6] y**	1.38 (1.01-1.90)	1.08 (0.50-2.34)	
**(6-7] y**	1.26 (0.88-1.82)	0.82 (0.30-2.27)	
**(7-8] y**	1.04 (0.64-1.71)	1.49 (0.64-3.48)	
**(8-9] y**	1.09 (0.59-2.01)	0.57 (0.08-4.26)	
**(9-10] y**	1.12 (0.54-2.32)	2.14 (0.73-6.30)	

CI, confidence interval.

Multivariable models were stratified by age at index date in 5-year interval and study areas, and adjusted for sex, marital status, education, tobacco smoking, alcohol consumption, level of total physical activity, body mass index, waist circumference, intake frequency of vegetables, fresh fruits, eggs, and red meat, self-reported health status, usage of antihypertensive or glucose-lowering drugs, major underlying conditions updated until the index date, and family history of cardiovascular disease.

Table S4. Sensitivity analyses for association of pneumonia hospitalisation with new-onset cardiovascular disease by restricting the exposed group to participants hospitalised for pneumonia during the first five years of follow-up

**Time interval after the index date**	**Adjusted hazard ratio (95% CI)**		
	**Ischaemic heart disease**	**Arrhythmia**	**Heart failure**
**0-90 d**	3.66 (2.48-5.38)	2.09 (0.60-7.28)	46.49 (9.36-230.90)
**91 d-1 y**	3.09 (2.45-3.90)	2.38 (1.21-4.71)	9.39 (5.26-16.74)
**(1-2] y**	2.69 (2.19-3.30)	3.43 (2.03-5.82)	5.07 (3.01-8.53)
**(2-3] y**	2.37 (1.91-2.95)	2.59 (1.48-4.54)	4.12 (2.41-7.05)
**(3-4] y**	2.43 (1.99-2.98)	2.09 (1.23-3.56)	2.76 (1.61-4.72)
**(4-5] y**	1.66 (1.32-2.09)	1.68 (1.00-2.83)	2.63 (1.57-4.42)
**(5-6] y**	1.79 (1.43-2.23)	2.24 (1.40-3.56)	2.77 (1.77-4.35)
**(6-7] y**	1.44 (1.12-1.85)	1.98 (1.22-3.22)	2.38 (1.44-3.96)
**(7-8] y**	1.17 (0.87-1.56)	2.45 (1.54-3.92)	3.83 (2.49-5.91)
**(8-9] y**	1.12 (0.79-1.60)	1.35 (0.71-2.60)	1.64 (0.78-3.42)
**(9-10] y**	1.19 (0.76-1.85)	1.08 (0.39-2.99)	1.84 (0.78-4.35)
	**Ischaemic stroke**	**Hemorrhagic stroke**	
**0-90 d**	2.72 (1.73-4.28)	2.94 (1.27-6.79)	
**91 d-1 y**	1.63 (1.21-2.18)	0.74 (0.34-1.60)	
**(1-2] y**	1.49 (1.15-1.94)	1.74 (1.09-2.80)	
**(2-3] y**	1.36 (1.04-1.77)	1.31 (0.75-2.27)	
**(3-4] y**	1.10 (0.83-1.45)	1.49 (0.91-2.44)	
**(4-5] y**	1.52 (1.20-1.93)	0.89 (0.50-1.61)	
**(5-6] y**	1.26 (0.98-1.63)	0.65 (0.32-1.33)	
**(6-7] y**	1.27 (0.98-1.66)	0.96 (0.54-1.73)	
**(7-8] y**	1.07 (0.78-1.46)	0.92 (0.47-1.81)	
**(8-9] y**	1.15 (0.81-1.62)	0.51 (0.16-1.61)	
**(9-10] y**	1.02 (0.64-1.63)	1.30 (0.56-3.02)	

CI, confidence interval.

The first two period after the index date in the primary analysis were merged into days 0-90 due to small sample size. Multivariable models were stratified by age at index date in 5-year interval and study areas, and adjusted for sex, marital status, education, tobacco smoking, alcohol consumption, level of total physical activity, body mass index, waist circumference, intake frequency of vegetables, fresh fruits, eggs, and red meat, frailty index, self-reported health status, usage of antihypertensive or glucose-lowering drugs, major underlying conditions updated until the index date, and family history of cardiovascular disease.

Table S5. Sensitivity analyses for association of pneumonia hospitalisation with new-onset cardiovascular disease by restricting the exposed group to participants without any other hospital admission in the previous 30 days

Time interval after the index date	Adjusted hazard ratio (95% CI)		
	**Ischaemic heart disease**	**Arrhythmia**	**Heart failure**
**0-30 d**	6.75 (5.24-8.69)	6.92 (3.73-12.82)	16.12 (7.72-33.67)
**31-90 d**	3.10 (2.50-3.84)	4.79 (3.02-7.62)	8.79 (4.99-15.50)
**91 d-1 y**	2.57 (2.31-2.87)	2.76 (2.12-3.60)	5.50 (4.24-7.13)
**(1-2] y**	2.51 (2.26-2.79)	2.84 (2.23-3.63)	3.97 (3.04-5.17)
**(2-3] y**	2.01 (1.79-2.27)	1.87 (1.41-2.49)	2.80 (2.08-3.75)
**(3-4] y**	2.01 (1.77-2.29)	1.98 (1.46-2.69)	2.03 (1.46-2.82)
**(4-5] y**	1.82 (1.56-2.12)	1.62 (1.11-2.36)	2.66 (1.89-3.75)
**(5-6] y**	1.74 (1.46-2.09)	1.97 (1.37-2.84)	2.90 (2.02-4.18)
**(6-7] y**	1.44 (1.15-1.80)	1.77 (1.14-2.74)	2.27 (1.44-3.59)
**(7-8] y**	1.46 (1.12-1.90)	2.79 (1.74-4.47)	3.60 (2.35-5.52)
**(8-9] y**	1.11 (0.79-1.58)	1.30 (0.68-2.49)	1.71 (0.82-3.59)
**(9-10] y**	1.20 (0.77-1.87)	1.01 (0.37-2.80)	1.65 (0.70-3.89)
	**Ischaemic stroke**	**Hemorrhagic stroke**	
**0-30 d**	2.32 (1.70-3.17)	3.65 (2.03-6.57)	
**31-90 d**	2.14 (1.68-2.72)	2.36 (1.45-3.85)	
**91 d-1 y**	1.44 (1.26-1.64)	1.24 (0.94-1.66)	
**(1-2] y**	1.63 (1.44-1.83)	1.28 (0.98-1.68)	
**(2-3] y**	1.53 (1.34-1.74)	1.04 (0.77-1.39)	
**(3-4] y**	1.52 (1.31-1.76)	1.25 (0.89-1.75)	
**(4-5] y**	1.52 (1.30-1.79)	0.88 (0.58-1.34)	
**(5-6] y**	1.35 (1.11-1.64)	0.77 (0.45-1.30)	
**(6-7] y**	1.31 (1.04-1.64)	1.00 (0.59-1.70)	
**(7-8] y**	1.25 (0.93-1.67)	1.00 (0.54-1.84)	
**(8-9] y**	1.09 (0.76-1.56)	0.30 (0.07-1.24)	
**(9-10] y**	1.06 (0.66-1.69)	1.17 (0.50-2.70)	

Multivariable models were stratified by age at index date in 5-year interval and study areas, and adjusted for sex, marital status, education, tobacco smoking, alcohol consumption, level of total physical activity, body mass index, waist circumference, intake frequency of vegetables, fresh fruits, eggs, and red meat, frailty index, self-reported health status, usage of antihypertensive or glucose-lowering drugs, major underlying conditions updated until the index date, and family history of cardiovascular disease.

Table S6. Sensitivity analyses for association of pneumonia hospitalisation with new-onset cardiovascular disease using competing risk model to account for the competing risk of death

**Time interval after the index date**	**Adjusted hazard ratio (95% CI)**		
	**Ischaemic heart disease**	**Arrhythmia**	**Heart failure**
**0-30 d**	5.76 (4.51-7.35)	5.03 (2.67-9.47)	18.50 (9.22-37.10)
**31-90 d**	3.22 (2.61-3.98)	12.80 (7.08-23.10)	9.72 (5.26-17.90)
**91 d-1 y**	2.67 (2.40-2.98)	3.02 (2.33-3.92)	5.78 (4.35-7.68)
**(1-2] y**	2.32 (2.09-2.57)	3.36 (2.63-4.28)	3.18 (2.45-4.14)
**(2-3] y**	1.95 (1.73-2.19)	2.02 (1.52-2.67)	3.23 (2.40-4.33)
**(3-4] y**	2.17 (1.91-2.46)	2.01 (1.49-2.70)	2.13 (1.53-2.97)
**(4-5] y**	1.90 (1.63-2.22)	1.56 (NA[a])	2.41 (1.73-3.37)
**(5-6] y**	1.72 (1.44-2.05)	1.78 (1.23-2.57)	2.95 (2.05-4.25)
**(6-7] y**	1.44 (1.16-1.80)	2.20 (1.43-3.40)	2.75 (1.72-4.39)
**(7-8] y**	1.45 (1.10-1.89)	2.89 (1.83-4.55)	4.31 (2.81-6.62)
**(8-9] y**	1.24 (0.87-1.77)	1.59 (0.80-3.13)	2.20 (1.02-4.75)
**(9-10] y**	1.27 (0.81-1.99)	0.91 (0.33-2.52)	2.16 (0.94-4.96)
	**Ischaemic stroke**	**Hemorrhagic stroke**	
**0-30 d**	2.59 (1.93-3.48)	3.52 (2.00-6.21)	
**31-90 d**	2.53 (2.00-3.20)	2.18 (1.39-3.42)	
**91 d-1 y**	1.53 (1.34-1.73)	1.21 (0.92-1.59)	
**(1-2] y**	1.63 (1.44-1.83)	1.38 (1.06-1.81)	
**(2-3] y**	1.65 (1.46-1.87)	1.23 (0.91-1.65)	
**(3-4] y**	1.48 (1.29-1.71)	1.06 (0.76-1.47)	
**(4-5] y**	1.47 (1.25-1.72)	0.97 (0.64-1.46)	
**(5-6] y**	1.39 (1.15-1.68)	0.68 (0.40-1.15)	
**(6-7] y**	1.29 (1.03-1.61)	1.04 (0.62-1.74)	
**(7-8] y**	1.11 (0.83-1.48)	1.19 (0.64-2.19)	
**(8-9] y**	1.27 (0.89-1.80)	0.45 (0.14-1.43)	
**(9-10] y**	1.19 (0.75-1.87)	1.06 (0.45-2.50)	

CI, confidence interval.

Multivariable models were adjusted for age at index date in 5-year interval, study area, sex, marital status, education, tobacco smoking, alcohol consumption, level of total physical activity, body mass index, waist circumference, intake frequency of vegetables, fresh fruits, eggs, and red meat, frailty index, self-reported health status, usage of antihypertensive or glucose-lowering drugs, major underlying conditions updated until the index date, and family history of cardiovascular disease.

[a] The estimate was not available due to the small number of cases.

Table S7. Adjusted hazard ratios (95% CIs) for incident ischaemic heart disease by subgroups after hospitalisation for pneumonia

Subgroup	Events/PYs (/1000) [a]		Time intervals after the index date [b]						P value for interaction
	Pneumonia cases	Matched controls	0-30 d	31-90 d	91 d-1 y	(1-3] y	(3-8] y	(8-10] y	
**Overall**	32.4	13.0	5.88 (4.64-7.45)	3.12 (2.54-3.84)	2.70 (2.42-3.00)	2.13 (1.97-2.31)	1.83 (1.70-1.98)	1.25 (0.95-1.64)	
**Age at index date, year**									0.02
<60	13.8	6.4	5.91 (3.53-9.90)	3.75 (2.34-6.00)	3.28 (2.62-4.11)	2.48 (2.11-2.91)	1.94 (1.69-2.24)	1.13 (0.70-1.83)	
≥60	41.6	18.9	5.86 (4.49-7.65)	2.99 (2.38-3.77)	2.56 (2.26-2.88)	2.04 (1.87-2.23)	1.79 (1.64-1.96)	1.31 (0.93-1.83)	
**Sex**									0.15
Male	32.5	12.4	7.08 (5.01-10.01)	3.19 (2.33-4.36)	2.72 (2.30-3.20)	2.22 (1.98-2.50)	1.92 (1.71-2.15)	1.38 (0.90-2.11)	
Female	32.3	13.4	4.94 (3.56-6.86)	3.04 (2.31-4.01)	2.67 (2.32-3.08)	2.07 (1.87-2.29)	1.76 (1.59-1.94)	1.15 (0.80-1.66)	
**Region**									<0.0001
Urban	29.0	14.2	5.06 (3.51-7.29)	3.45 (2.55-4.65)	1.97 (1.65-2.37)	1.88 (1.66-2.14)	1.73 (1.54-1.95)	1.33 (0.89-1.98)	
Rural	34.0	12.0	6.57 (4.80-9.00)	2.87 (2.16-3.81)	3.28 (2.86-3.75)	2.31 (2.09-2.55)	1.91 (1.73-2.11)	1.20 (0.82-1.75)	
**Educational attainment**									0.09
Illiterate or primary school	32.4	12.9	6.56 (4.90-8.77)	2.90 (2.22-3.78)	2.98 (2.61-3.40)	2.16 (1.96-2.37)	1.81 (1.65-1.99)	1.16 (0.81-1.66)	
Middle school or higher	32.3	13.0	4.70 (3.08-7.17)	3.48 (2.50-4.83)	2.23 (1.85-2.69)	2.07 (1.81-2.36)	1.87 (1.65-2.12)	1.45 (0.93-2.24)	
**Prevalent hypertension** [c]									0.08
No	27.2	10.3	5.87 (4.22-8.17)	3.09 (2.26-4.23)	2.75 (2.36-3.20)	2.40 (2.16-2.68)	1.83 (1.64-2.04)	1.21 (0.82-1.79)	
Yes	39.1	16.1	5.89 (4.19-8.28)	3.15 (2.39-4.16)	2.64 (2.28-3.07)	1.91 (1.71-2.13)	1.83 (1.65-2.04)	1.26 (0.85-1.87)	
**Prevalent diabetes** [c]									0.45
No	30.9	12.3	5.40 (4.15-7.02)	3.04 (2.43-3.79)	2.78 (2.47-3.12)	2.18 (2.01-2.37)	1.83 (1.69-1.99)	1.30 (0.97-1.73)	
Yes	47.0	19.3	8.27 (4.63-14.75)	3.48 (1.94-6.24)	2.20 (1.67-2.91)	1.82 (1.46-2.27)	1.81 (1.44-2.26)	0.81 (0.33-2.03)	
**Prevalent COPD** [c]									0.77

Subgroup	Events/PYs (/1000) [a]		Time intervals after the index date [b]						P value for interaction
	Pneumonia cases	Matched controls	0-30 d	31-90 d	91 d-1 y	(1-3] y	(3-8] y	(8-10] y	
No	30.9	12.6	6.05 (4.64-7.89)	3.44 (2.74-4.33)	2.77 (2.45-3.13)	2.11 (1.93-2.31)	1.86 (1.71-2.03)	1.27 (0.94-1.73)	
Yes	37.1	16.4	5.27 (3.10-8.96)	2.14 (1.32-3.48)	2.43 (1.93-3.05)	2.21 (1.88-2.59)	1.72 (1.46-2.03)	1.09 (0.58-2.05)	
**Tobacco smoking**									0.95
Nonsmoker	31.3	12.2	4.96 (3.67-6.71)	3.24 (2.52-4.16)	2.65 (2.33-3.02)	2.10 (1.91-2.32)	1.88 (1.71-2.06)	1.09 (0.76-1.56)	
Current smoker [d]	34.6	15.2	7.81 (5.28-11.54)	2.88 (1.99-4.18)	2.80 (2.32-3.38)	2.21 (1.94-2.52)	1.73 (1.51-1.97)	1.52 (0.98-2.34)	
**Alcohol drinking**									0.0063
Non-excessive drinker	31.5	12.9	5.58 (4.30-7.25)	3.04 (2.43-3.80)	2.60 (2.31-2.91)	2.07 (1.90-2.25)	1.81 (1.67-1.96)	1.25 (0.92-1.68)	
Excessive drinker [e]	37.5	13.6	7.21 (4.06-12.80)	3.50 (1.96-6.24)	3.52 (2.65-4.67)	2.54 (2.08-3.09)	2.04 (1.66-2.51)	1.29 (0.62-2.68)	
**BMI, kg/m^2**									0.03
Underweight, <18.5	33.8	12.1	6.06 (2.30-15.93)	2.42 (1.06-5.52)	3.57 (2.38-5.35)	2.03 (1.54-2.69)	2.30 (1.77-3.00)	1.89 (0.80-4.46)	
Normal or overweight, 18.5-27.9	31.2	12.4	5.78 (4.43-7.54)	3.44 (2.74-4.31)	2.66 (2.36-3.00)	2.18 (2.00-2.38)	1.78 (1.63-1.94)	1.26 (0.92-1.71)	
Obesity, ≥28.0	42.0	17.2	6.36 (3.41-11.85)	1.80 (0.91-3.59)	2.49 (1.86-3.34)	1.90 (1.51-2.40)	1.95 (1.57-2.42)	0.71 (0.26-1.96)	
**Waist circumference, cm**									0.35
Male<90, female<85	30.2	11.9	5.79 (4.33-7.74)	3.48 (2.71-4.46)	2.80 (2.46-3.18)	2.13 (1.94-2.34)	1.88 (1.71-2.05)	1.23 (0.88-1.71)	
Male≥90, female≥85	39.5	15.7	6.07 (4.03-9.13)	2.48 (1.69-3.64)	2.48 (2.05-3.01)	2.14 (1.86-2.46)	1.72 (1.49-1.98)	1.36 (0.83-2.23)	
**Family history of CVD [f]**									0.01
No	32.2	12.5	6.11 (4.70-7.94)	3.46 (2.74-4.37)	2.82 (2.50-3.18)	2.14 (1.96-2.33)	1.89 (1.73-2.06)	1.33 (0.99-1.79)	
Yes	33.3	14.8	4.88 (2.81-8.48)	2.18 (1.38-3.44)	2.32 (1.83-2.94)	2.12 (1.78-2.52)	1.64 (1.39-1.94)	0.80 (0.37-1.73)	

CI, confidence interval; PYs, person-years; COPD: chronic obstructive pulmonary disease; CVD, cardiovascular diseases.

[a] Data were adjusted for age at index date, sex, and study area where appropriate.

[b] Please refer to Figure 2 for covariates adjusted in the models, except in case of stratifying by corresponding variable.

[c] The disease status of hypertension, diabetes, and COPD were updated until the index date.

[d] Participants who quit smoking because of illness were classified as current daily smokers.

[e] Excessive drinker was defined as those who drank ≥30 g of pure alcohol per day, or having stopped drinking.

[f] A CVD family history was defined as having at least one first-degree relative (biological father, mother, and siblings) with heart disease or stroke.

Table S8. Adjusted hazard ratios (95% CIs) for incident arrhythmia by subgroups after hospitalisation for pneumonia

Subgroup	Events/PYs (/1000) [a]		Time intervals after the index date [b]						P value for interaction
	Pneumonia cases	Matched controls	0-30 d	31-90 d	91 d-1 y	(1-3] y	(3-8] y	(8-10] y	
**Overall**	6.2	2.0	5.00 (2.83-8.82)	12.32 (7.03-21.61)	3.08 (2.38-3.99)	2.68 (2.24-3.21)	1.92 (1.63-2.27)	1.31 (0.75-2.27)	
**Age at index date, year**									0.48
<60	2.8	1.0	7.54 (2.87-19.82)	15.99 (4.72-54.14)	3.51 (2.10-5.88)	3.32 (2.25-4.88)	1.73 (1.21-2.47)	1.51 (0.59-3.82)	
≥60	7.7	2.8	4.11 (2.06-8.16)	11.52 (6.15-21.61)	2.96 (2.20-3.98)	2.55 (2.08-3.12)	2.00 (1.66-2.41)	1.21 (0.61-2.41)	
**Sex**									0.0002
Male	7.7	2.0	5.98 (2.76-12.98)	25.85 (10.86-61.52)	4.17 (2.83-6.14)	2.87 (2.19-3.75)	2.62 (2.09-3.30)	1.23 (0.53-2.84)	
Female	5.1	2.0	4.00 (1.73-9.26)	5.62 (2.41-13.13)	2.46 (1.74-3.48)	2.55 (2.00-3.26)	1.45 (1.13-1.85)	1.36 (0.65-2.83)	
**Region**									0.01
Urban	4.9	2.1	4.36 (1.87-10.18)	13.33 (5.67-31.29)	2.12 (1.32-3.40)	2.55 (1.88-3.45)	1.54 (1.17-2.04)	1.04 (0.42-2.59)	
Rural	7.1	1.9	5.66 (2.60-12.28)	11.66 (5.54-24.55)	3.73 (2.73-5.10)	2.77 (2.21-3.47)	2.22 (1.80-2.73)	1.52 (0.76-3.06)	
**Educational attainment**									0.69
Illiterate or primary school	6.0	1.9	4.77 (2.39-9.52)	9.66 (4.74-19.69)	3.36 (2.44-4.63)	2.64 (2.12-3.29)	1.98 (1.62-2.42)	1.21 (0.59-2.51)	
Middle school or higher	6.9	2.2	5.44 (2.01-14.71)	18.31 (7.26-46.20)	2.54 (1.63-3.96)	2.78 (2.02-3.81)	1.83 (1.36-2.46)	1.51 (0.65-3.51)	
**Prevalent hypertension** [c]									0.43
No	5.7	1.8	5.65 (2.60-12.26)	9.43 (4.09-21.77)	3.71 (2.64-5.22)	2.76 (2.16-3.53)	1.90 (1.50-2.41)	1.35 (0.65-2.82)	
Yes	6.9	2.3	4.30 (1.87-9.92)	15.97 (7.39-34.49)	2.47 (1.66-3.67)	2.56 (1.96-3.35)	1.96 (1.55-2.47)	1.27 (0.55-2.92)	
**Prevalent diabetes** [c]									0.44
No	6.2	2.0	4.75 (2.66-8.51)	11.87 (6.54-21.54)	3.07 (2.33-4.05)	2.69 (2.22-3.26)	2.00 (1.68-2.38)	1.34 (0.75-2.37)	
Yes	6.5	2.3	NA[g]	16.85 (2.86-99.32)	3.07 (1.47-6.40)	2.69 (1.58-4.60)	1.35 (0.77-2.39)	1.35 (0.17-10.62)	
**Prevalent COPD** [c]									0.68
No	6.0	1.9	6.62 (3.58-12.23)	12.43 (6.72-23.02)	3.23 (2.40-4.34)	2.72 (2.21-3.36)	1.80 (1.48-2.20)	1.75 (1.00-3.06)	

Subgroup	Events/PYs (/1000) [a]		Time intervals after the index date [b]						P value for interaction
	Pneumonia cases	Matched controls	0-30 d	31-90 d	91 d-1 y	(1-3] y	(3-8] y	(8-10] y	
Yes	6.9	2.6	1.57 (0.38-6.58)	16.27 (3.42-77.47)	2.70 (1.60-4.57)	2.56 (1.78-3.67)	2.20 (1.60-3.01)	NA[g]	
**Tobacco smoking**									0.01
Nonsmoker	6.0	1.9	4.88 (2.48-9.61)	8.90 (4.40-17.99)	2.58 (1.85-3.61)	2.68 (2.12-3.37)	1.63 (1.31-2.03)	1.39 (0.72-2.67)	
Current smoker [d]	6.5	2.1	5.49 (1.92-15.74)	22.23 (7.96-62.09)	4.03 (2.67-6.07)	2.71 (2.02-3.62)	2.56 (1.98-3.33)	1.10 (0.39-3.10)	
**Alcohol drinking**									0.03
Non-excessive drinker	6.2	2.0	5.20 (2.72-9.94)	11.99 (6.38-22.51)	2.63 (1.97-3.53)	2.72 (2.23-3.30)	1.73 (1.43-2.09)	1.57 (0.90-2.75)	
Excessive drinker [e]	6.3	2.0	3.99 (1.23-12.95)	13.77 (3.97-47.73)	6.05 (3.37-10.87)	2.46 (1.53-3.96)	2.95 (2.05-4.26)	NA[g]	
**BMI, kg/m^2**									0.72
Underweight, <18.5	6.2	2.3	3.85 (0.62-23.84)	NA[g]	5.88 (2.58-13.37)	1.55 (0.79-3.05)	2.16 (1.21-3.87)	1.61 (0.20-13.24)	
Normal or overweight, 18.5-27.9	6.1	1.9	4.25 (2.22-8.15)	11.37 (6.07-21.32)	2.99 (2.25-3.97)	2.85 (2.33-3.47)	1.92 (1.60-2.31)	1.27 (0.68-2.36)	
Obesity, ≥28.0	7.7	2.2	13.28 (2.81-62.70)	35.36 (6.99-178.82)	1.64 (0.55-4.89)	2.89 (1.62-5.16)	1.69 (0.97-2.93)	1.99 (0.45-8.87)	
**Waist circumference, cm**									0.21
Male<90, female<85	6.2	2.0	3.95 (2.04-7.66)	11.74 (5.80-23.75)	3.53 (2.66-4.68)	2.75 (2.24-3.37)	1.97 (1.63-2.39)	1.43 (0.76-2.67)	
Male≥90, female≥85	6.3	2.1	9.52 (3.04-29.80)	13.80 (5.41-35.15)	1.56 (0.78-3.12)	2.42 (1.63-3.57)	1.79 (1.28-2.50)	1.11 (0.34-3.60)	
**Family history of CVD [f]**									0.12
No	6.3	2.0	6.85 (3.58-13.13)	10.70 (5.78-19.82)	3.29 (2.50-4.34)	2.67 (2.18-3.25)	2.00 (1.66-2.39)	1.62 (0.91-2.89)	
Yes	5.9	2.2	1.74 (0.46-6.65)	23.82 (5.98-94.90)	2.10 (1.02-4.30)	2.76 (1.79-4.26)	1.64 (1.09-2.47)	0.41 (0.05-2.99)	

CI, confidence interval; PYs, person-years; COPD: chronic obstructive pulmonary disease; CVD, cardiovascular diseases.

[a] Data were adjusted for age at index date, sex, and study area where appropriate.

[b] Please refer to Figure 2 for covariates adjusted in the models, except in case of stratifying by corresponding variable.

[c] The disease status of hypertension, diabetes, and COPD were updated until the index date.

[d] Participants who quit smoking because of illness were classified as current daily smokers.

[e] Excessive drinker was defined as those who drank ≥30 g of pure alcohol per day, or having stopped drinking.

[f] A CVD family history was defined as having at least one first-degree relative (biological father, mother, and siblings) with heart disease or stroke.

[g] Estimates were not available due to the small number of cases.

Table S9. Adjusted hazard ratios (95% CIs) for incident heart failure by subgroups after hospitalisation for pneumonia

Subgroup	Events/PYs (/1000) [a]		Time intervals after the index date [b]						P value for interaction
	Pneumonia cases	Matched controls	0-30 d	31-90 d	91 d-1 y	(1-3] y	(3-8] y	(8-10] y	
**Overall**	7.3	1.5	19.29 (9.48-39.23)	9.81 (5.63-17.08)	5.85 (4.52-7.59)	3.20 (2.65-3.87)	2.68 (2.27-3.17)	1.91 (1.09-3.37)	
**Age at index date, year**									0.0020
<60	2.1	0.3	27.93 (5.34-146.08)	77.80 (9.21-657.05)	17.21 (7.51-39.41)	5.30 (3.00-9.38)	3.78 (2.50-5.72)	0.83 (0.19-3.58)	
≥60	9.6	2.5	17.44 (7.96-38.19)	7.36 (4.08-13.27)	5.14 (3.90-6.77)	3.02 (2.47-3.69)	2.52 (2.09-3.02)	2.32 (1.26-4.28)	
**Sex**									0.61
Male	8.3	1.6	24.33 (9.53-62.07)	10.38 (4.54-23.75)	5.12 (3.56-7.38)	3.09 (2.33-4.10)	2.79 (2.19-3.56)	2.05 (0.92-4.56)	
Female	6.5	1.4	14.11 (4.65-42.82)	9.31 (4.35-19.93)	6.86 (4.74-9.92)	3.27 (2.53-4.23)	2.57 (2.04-3.25)	1.78 (0.79-3.98)	
**Region**									0.64
Urban	5.6	1.1	48.49 (10.54-223.04)	9.02 (3.55-22.88)	5.75 (3.72-8.86)	4.01 (2.87-5.59)	2.27 (1.69-3.05)	2.28 (0.96-5.41)	
Rural	8.3	1.7	13.21 (5.83-29.91)	10.26 (5.16-20.41)	5.93 (4.29-8.19)	2.88 (2.28-3.62)	2.95 (2.40-3.62)	1.75 (0.83-3.70)	
**Educational attainment**									0.64
Illiterate or primary school	7.7	1.6	17.15 (7.74-38.00)	7.31 (3.87-13.82)	5.63 (4.17-7.60)	3.35 (2.72-4.11)	2.71 (2.24-3.27)	1.87 (0.98-3.55)	
Middle school or higher	5.9	1.2	29.27 (5.96-143.83)	26.11 (7.08-96.33)	6.76 (4.06-11.26)	2.37 (1.44-3.93)	2.65 (1.84-3.81)	1.94 (0.57-6.59)	
**Prevalent hypertension** [c]									0.65
No	6.0	1.2	12.06 (4.95-29.38)	6.16 (2.63-14.46)	7.47 (4.98-11.19)	2.75 (2.06-3.67)	2.73 (2.14-3.48)	2.32 (1.01-5.32)	
Yes	8.7	1.8	36.78 (10.49-128.92)	13.59 (6.43-28.72)	4.98 (3.53-7.02)	3.63 (2.82-4.67)	2.56 (2.03-3.24)	1.75 (0.80-3.84)	
**Prevalent diabetes** [c]									0.63
No	6.8	1.4	11.50 (5.19-25.46)	8.60 (4.66-15.87)	5.91 (4.47-7.81)	3.25 (2.65-3.99)	2.65 (2.21-3.17)	2.32 (1.31-4.12)	
Yes	11.3	2.6	102.77 (12.86-821.47)	15.95 (4.00-63.54)	5.61 (2.79-11.26)	3.03 (1.80-5.10)	2.84 (1.79-4.52)	NA[g]	

Subgroup	Events/PYs (/1000) [a]		Time intervals after the index date [b]						P value for interaction
	Pneumonia cases	Matched controls	0-30 d	31-90 d	91 d-1 y	(1-3] y	(3-8] y	(8-10] y	
**Prevalent COPD [c]**									0.22
No	5.9	1.3	26.93 (11.19-64.81)	16.27 (8.28-31.98)	6.99 (5.10-9.59)	3.46 (2.72-4.39)	2.46 (1.98-3.06)	1.72 (0.85-3.45)	
Yes	10.8	2.7	9.38 (2.91-30.19)	3.49 (1.38-8.84)	4.16 (2.68-6.45)	2.87 (2.10-3.94)	3.08 (2.35-4.03)	2.22 (0.80-6.13)	
**Tobacco smoking**									0.93
Nonsmoker	6.8	1.4	13.24 (5.44-32.24)	9.73 (4.91-19.28)	5.50 (3.90-7.74)	3.60 (2.82-4.60)	2.69 (2.16-3.34)	1.52 (0.73-3.19)	
Current smoker [d]	8.1	1.7	35.91 (10.15-127.13)	9.77 (3.79-25.18)	6.19 (4.16-9.21)	2.68 (1.98-3.63)	2.64 (2.03-3.45)	2.82 (1.16-6.85)	
**Alcohol drinking**									0.48
Non-excessive drinker	7.1	1.5	15.48 (7.38-32.48)	8.39 (4.58-15.40)	6.08 (4.58-8.06)	3.23 (2.62-3.98)	2.48 (2.06-3.00)	2.13 (1.16-3.94)	
Excessive drinker [e]	7.9	1.6	NA[g]	25.29 (5.20-123.06)	4.54 (2.39-8.62)	2.99 (1.90-4.72)	3.75 (2.59-5.45)	1.05 (0.24-4.61)	
**BMI, kg/m^2**									0.51
Underweight, <18.5	10.0	2.2	8.54 (0.57-128.24)	NA[g]	3.80 (1.77-8.14)	1.96 (1.12-3.44)	2.34 (1.44-3.79)	3.95 (0.93-16.72)	
Normal or overweight, 18.5-27.9	6.9	1.4	20.96 (9.59-45.79)	9.14 (5.04-16.58)	6.15 (4.61-8.21)	3.24 (2.61-4.02)	2.77 (2.29-3.35)	1.73 (0.86-3.49)	
Obesity, ≥28.0	8.7	2.1	16.22 (1.63-161.37)	21.36 (3.48-131.08)	5.09 (1.91-13.55)	6.45 (3.46-12.01)	2.03 (1.15-3.59)	1.64 (0.38-7.13)	
**Waist circumference, cm**									0.54
Male<90, female<85	6.9	1.4	14.91 (6.67-33.33)	10.16 (5.35-19.28)	5.26 (3.87-7.13)	3.15 (2.54-3.92)	2.71 (2.23-3.30)	2.11 (1.07-4.16)	
Male≥90, female≥85	8.6	1.8	38.44 (8.10-182.42)	9.17 (3.02-27.86)	7.58 (4.65-12.37)	3.29 (2.22-4.87)	2.61 (1.87-3.64)	1.65 (0.58-4.68)	
**Family history of CVD [f]**									0.28
No	7.5	1.5	19.50 (8.89-42.74)	7.56 (4.17-13.73)	5.41 (4.10-7.14)	3.36 (2.74-4.12)	2.80 (2.34-3.36)	2.32 (1.31-4.13)	
Yes	5.9	1.6	18.53 (3.48-98.55)	55.60 (6.60-468.16)	10.37 (4.95-21.69)	2.39 (1.39-4.12)	2.14 (1.36-3.37)	NA[g]	

CI, confidence interval; PYs, person-years; COPD: chronic obstructive pulmonary disease; CVD, cardiovascular diseases.

[a] Data were adjusted for age at index date, sex, and study area where appropriate.

[b] Please refer to Figure 2 for covariates adjusted in the models, except in case of stratifying by corresponding variable.

[c] The disease status of hypertension, diabetes, and COPD were updated until the index date.

[d] Participants who quit smoking because of illness were classified as current daily smokers.

[e] Excessive drinker was defined as those who drank ≥30 g of pure alcohol per day, or having stopped drinking.

[f] A CVD family history was defined as having at least one first-degree relative (biological father, mother, and siblings) with heart disease or stroke.

[g] Estimates were not available due to the small number of cases.

Table S10. Adjusted hazard ratios (95% CIs) for incident ischaemic stroke by subgroups after hospitalisation for pneumonia

Subgroup	Events/PYs (/1000) [a]		Time intervals after the index date [b]						P value for interaction
	Pneumonia cases	Matched controls	0-30 d	31-90 d	91 d-1 y	(1-3] y	(3-8] y	(8-10] y	
**Overall**	23.6	14.6	2.56 (1.91-3.44)	2.52 (1.99-3.20)	1.53 (1.35-1.74)	1.60 (1.46-1.74)	1.38 (1.27-1.50)	1.25 (0.94-1.65)	
**Age at index date, year**									0.22
<60	9.3	7.3	2.65 (1.34-5.26)	3.05 (1.82-5.12)	1.38 (1.01-1.88)	1.62 (1.34-1.96)	1.28 (1.09-1.51)	1.35 (0.88-2.08)	
≥60	30.4	21.0	2.55 (1.85-3.53)	2.41 (1.84-3.14)	1.57 (1.37-1.80)	1.59 (1.45-1.75)	1.43 (1.30-1.57)	1.18 (0.81-1.70)	
**Sex**									0.26
Male	25.6	15.3	2.54 (1.64-3.93)	2.89 (2.09-4.00)	1.43 (1.18-1.73)	1.59 (1.40-1.80)	1.55 (1.37-1.74)	1.08 (0.69-1.70)	
Female	21.9	14.0	2.62 (1.76-3.89)	2.18 (1.54-3.08)	1.62 (1.37-1.93)	1.60 (1.42-1.79)	1.26 (1.12-1.41)	1.35 (0.94-1.94)	
**Region**									0.92
Urban	26.8	16.1	2.27 (1.50-3.43)	2.82 (2.02-3.94)	1.63 (1.36-1.96)	1.58 (1.39-1.79)	1.38 (1.23-1.55)	0.98 (0.63-1.55)	
Rural	21.0	13.4	2.93 (1.92-4.45)	2.27 (1.62-3.18)	1.45 (1.22-1.73)	1.61 (1.43-1.80)	1.38 (1.23-1.54)	1.49 (1.04-2.13)	
**Educational attainment**									0.57
Illiterate or primary school	24.0	15.0	3.07 (2.13-4.43)	2.52 (1.86-3.41)	1.64 (1.41-1.92)	1.57 (1.41-1.74)	1.38 (1.25-1.53)	1.40 (1.00-1.97)	
Middle school or higher	22.6	13.8	1.91 (1.15-3.16)	2.51 (1.71-3.69)	1.32 (1.06-1.66)	1.66 (1.43-1.91)	1.39 (1.22-1.60)	0.98 (0.59-1.62)	
**Prevalent hypertension** [c]									0.0049
No	18.8	10.6	2.79 (1.77-4.38)	2.41 (1.68-3.47)	1.73 (1.43-2.09)	1.76 (1.55-1.99)	1.46 (1.29-1.64)	1.41 (0.96-2.05)	
Yes	29.6	19.4	2.44 (1.65-3.59)	2.57 (1.87-3.52)	1.41 (1.19-1.67)	1.48 (1.32-1.66)	1.31 (1.17-1.47)	1.06 (0.69-1.61)	
**Prevalent diabetes** [c]									0.02
No	22.5	13.7	2.75 (2.01-3.76)	2.49 (1.91-3.23)	1.61 (1.41-1.84)	1.65 (1.51-1.81)	1.39 (1.28-1.52)	1.13 (0.83-1.54)	
Yes	32.8	24.3	1.72 (0.73-4.04)	2.40 (1.38-4.16)	1.10 (0.76-1.59)	1.27 (1.00-1.62)	1.29 (1.04-1.61)	2.13 (1.07-4.25)	
**Prevalent COPD** [c]									0.89

Subgroup	Events/PYs (/1000) [a]		Time intervals after the index date [b]						P value for interaction
	Pneumonia cases	Matched controls	0-30 d	31-90 d	91 d-1 y	(1-3] y	(3-8] y	(8-10] y	
No	24.1	14.6	2.58 (1.86-3.58)	2.25 (1.72-2.96)	1.44 (1.25-1.67)	1.59 (1.44-1.75)	1.44 (1.32-1.57)	1.26 (0.92-1.72)	
Yes	21.8	14.3	2.31 (1.16-4.60)	4.11 (2.37-7.11)	1.83 (1.40-2.40)	1.67 (1.37-2.03)	1.16 (0.95-1.42)	1.37 (0.71-2.68)	
**Tobacco smoking**									0.57
Nonsmoker	22.8	14.0	2.63 (1.84-3.75)	2.49 (1.85-3.35)	1.51 (1.29-1.77)	1.66 (1.50-1.85)	1.30 (1.18-1.44)	1.07 (0.74-1.54)	
Current smoker [d]	24.9	15.9	2.52 (1.50-4.23)	2.58 (1.74-3.81)	1.58 (1.28-1.96)	1.47 (1.26-1.71)	1.56 (1.36-1.79)	1.66 (1.06-2.58)	
**Alcohol drinking**									0.71
Non-excessive drinker	23.3	14.3	2.51 (1.82-3.45)	2.60 (2.02-3.36)	1.49 (1.30-1.71)	1.60 (1.45-1.75)	1.37 (1.25-1.49)	1.27 (0.94-1.71)	
Excessive drinker [e]	25.3	16.8	2.63 (1.25-5.50)	2.15 (1.11-4.14)	1.75 (1.26-2.42)	1.62 (1.29-2.03)	1.49 (1.20-1.86)	1.07 (0.49-2.34)	
**BMI, kg/m^2**									0.17
Underweight, <18.5	18.4	9.7	2.82 (0.49-16.12)	3.45 (1.50-7.96)	1.68 (1.03-2.76)	2.05 (1.43-2.94)	1.69 (1.21-2.36)	0.66 (0.09-5.05)	
Normal or overweight, 18.5-27.9	23.5	14.3	2.55 (1.85-3.52)	2.46 (1.88-3.22)	1.61 (1.40-1.84)	1.56 (1.42-1.72)	1.38 (1.26-1.50)	1.34 (0.99-1.81)	
Obesity, ≥28.0	29.6	19.2	2.77 (1.23-6.21)	2.38 (1.23-4.60)	1.01 (0.67-1.53)	1.70 (1.33-2.19)	1.26 (0.98-1.62)	1.05 (0.46-2.43)	
**Waist circumference, cm**									0.10
Male<90, female<85	22.2	13.4	2.56 (1.79-3.64)	2.63 (1.98-3.49)	1.65 (1.42-1.91)	1.60 (1.44-1.78)	1.44 (1.31-1.59)	1.30 (0.94-1.81)	
Male≥90, female≥85	27.8	17.7	2.58 (1.53-4.36)	2.23 (1.43-3.45)	1.31 (1.03-1.66)	1.58 (1.35-1.85)	1.24 (1.07-1.45)	1.15 (0.67-1.99)	
**Family history of CVD [f]**									0.94
No	22.4	14.0	2.81 (2.03-3.90)	2.38 (1.81-3.13)	1.60 (1.39-1.84)	1.62 (1.47-1.78)	1.32 (1.20-1.45)	1.24 (0.90-1.70)	
Yes	28.8	16.8	1.80 (0.90-3.59)	3.06 (1.91-4.90)	1.31 (0.98-1.76)	1.52 (1.26-1.83)	1.59 (1.35-1.88)	1.27 (0.68-2.35)	

CI, confidence interval; PYs, person-years; COPD: chronic obstructive pulmonary disease; CVD, cardiovascular diseases.

[a] Data were adjusted for age at index date, sex, and study area where appropriate.

[b] Please refer to Figure 2 for covariates adjusted in the models, except in case of stratifying by corresponding variable.

[c] The disease status of hypertension, diabetes, and COPD were updated until the index date.

[d] Participants who quit smoking because of illness were classified as current daily smokers.

[e] Excessive drinker was defined as those who drank ≥30 g of pure alcohol per day, or having stopped drinking.

[f] A CVD family history was defined as having at least one first-degree relative (biological father, mother, and siblings) with heart disease or stroke.

Table S11. Adjusted hazard ratios (95% CIs) for incident hemorrhagic stroke by subgroups after hospitalisation for pneumonia

Subgroup	Events/PYs (/1000) [a]		Time intervals after the index date [b]						P value for interaction
	Pneumonia cases	Matched controls	0-30 d	31-90 d	91 d-1 y	(1-3] y	(3-8] y	(8-10] y	
**Overall**	4.5	3.1	3.82 (2.15-6.78)	2.21 (1.43-3.44)	1.29 (0.98-1.69)	1.30 (1.07-1.58)	0.96 (0.79-1.17)	0.74 (0.38-1.46)	
**Age at index date, year**									0.03
<60	1.8	1.2	10.60 (3.44-32.70)	1.91 (0.66-5.53)	1.57 (0.78-3.15)	2.22 (1.48-3.33)	0.98 (0.63-1.51)	1.47 (0.58-3.74)	
≥60	5.7	4.8	2.80 (1.43-5.48)	2.30 (1.41-3.73)	1.25 (0.93-1.67)	1.14 (0.91-1.42)	0.95 (0.76-1.19)	0.46 (0.17-1.25)	
**Sex**									0.73
Male	5.4	3.6	4.71 (2.05-10.84)	2.70 (1.58-4.62)	1.34 (0.93-1.93)	1.26 (0.96-1.65)	0.94 (0.71-1.25)	0.90 (0.39-2.06)	
Female	3.7	2.7	3.36 (1.52-7.39)	1.53 (0.70-3.34)	1.27 (0.84-1.92)	1.34 (1.01-1.76)	0.97 (0.73-1.28)	0.57 (0.18-1.81)	
**Region**									0.26
Urban	2.9	1.8	2.43 (0.68-8.73)	2.80 (1.17-6.71)	1.49 (0.88-2.53)	1.69 (1.19-2.41)	0.95 (0.64-1.41)	0.62 (0.15-2.57)	
Rural	5.6	4.0	4.39 (2.28-8.45)	2.05 (1.23-3.42)	1.23 (0.90-1.69)	1.18 (0.93-1.49)	0.96 (0.76-1.21)	0.78 (0.36-1.67)	
**Educational attainment**									0.02
Illiterate or primary school	4.6	3.3	3.92 (2.02-7.60)	2.19 (1.27-3.77)	1.28 (0.95-1.73)	1.14 (0.90-1.43)	0.90 (0.71-1.13)	0.74 (0.35-1.60)	
Middle school or higher	4.2	2.5	3.45 (1.06-11.17)	2.30 (1.09-4.86)	1.33 (0.71-2.47)	1.99 (1.38-2.87)	1.22 (0.81-1.83)	0.75 (0.18-3.12)	
**Prevalent hypertension** [c]									0.11
No	2.7	1.6	6.50 (2.38-17.76)	3.45 (1.63-7.31)	1.75 (1.13-2.69)	1.44 (1.03-2.00)	0.83 (0.58-1.19)	0.52 (0.16-1.68)	
Yes	6.7	4.8	3.09 (1.50-6.35)	1.82 (1.05-3.18)	1.07 (0.75-1.52)	1.24 (0.97-1.57)	1.02 (0.80-1.29)	0.84 (0.37-1.92)	
**Prevalent diabetes** [c]									0.67
No	4.4	3.0	3.73 (2.05-6.80)	2.32 (1.44-3.75)	1.34 (1.01-1.77)	1.31 (1.07-1.60)	0.93 (0.75-1.15)	0.71 (0.35-1.46)	
Yes	5.6	4.5	5.71 (0.73-44.68)	1.70 (0.54-5.37)	0.91 (0.35-2.37)	1.19 (0.62-2.28)	1.15 (0.66-2.00)	0.84 (0.10-6.78)	

Subgroup	Events/PYs (/1000) [a]		Time intervals after the index date [b]						P value for interaction
	Pneumonia cases	Matched controls	0-30 d	31-90 d	91 d-1 y	(1-3] y	(3-8] y	(8-10] y	
**Prevalent COPD [c]**									0.36
No	4.4	3.0	4.65 (2.35-9.17)	2.46 (1.52-3.98)	1.25 (0.90-1.72)	1.28 (1.02-1.61)	1.02 (0.82-1.28)	1.06 (0.54-2.09)	
Yes	4.7	3.6	2.19 (0.78-6.12)	1.56 (0.54-4.52)	1.38 (0.82-2.31)	1.37 (0.94-1.99)	0.80 (0.51-1.23)	NA[g]	
**Tobacco smoking**									0.47
Nonsmoker	4.4	2.9	3.23 (1.56-6.71)	2.13 (1.14-3.99)	1.35 (0.94-1.94)	1.44 (1.12-1.84)	1.04 (0.81-1.33)	0.48 (0.15-1.53)	
Current smoker [d]	4.6	3.5	5.54 (2.16-14.22)	2.31 (1.24-4.28)	1.23 (0.81-1.85)	1.13 (0.83-1.55)	0.83 (0.60-1.15)	1.11 (0.48-2.58)	
**Alcohol drinking**									0.20
Non-excessive drinker	4.4	2.9	4.22 (2.30-7.74)	2.24 (1.38-3.65)	1.45 (1.08-1.96)	1.33 (1.08-1.65)	0.99 (0.79-1.23)	0.52 (0.21-1.26)	
Excessive drinker [e]	5.0	4.3	2.15 (0.38-11.98)	2.00 (0.72-5.54)	0.80 (0.42-1.53)	1.13 (0.71-1.78)	0.80 (0.50-1.27)	1.30 (0.45-3.71)	
**BMI, kg/m^2**									0.53
Underweight, <18.5	5.0	3.2	4.29 (0.84-21.94)	5.50 (1.60-18.98)	0.88 (0.30-2.56)	1.27 (0.73-2.21)	0.88 (0.45-1.71)	NA[g]	
Normal or overweight, 18.5-27.9	4.4	3.0	4.15 (2.21-7.80)	2.07 (1.26-3.41)	1.36 (1.01-1.81)	1.28 (1.03-1.60)	0.99 (0.80-1.22)	0.91 (0.46-1.80)	
Obesity, ≥28.0	4.0	3.7	NA[g]	0.97 (0.12-7.70)	0.92 (0.27-3.05)	1.44 (0.78-2.67)	0.70 (0.30-1.59)	0.00 (0.00-Inf)	
**Waist circumference, cm**									0.87
Male<90, female<85	4.4	3.0	3.34 (1.70-6.56)	2.55 (1.58-4.12)	1.36 (1.01-1.84)	1.19 (0.95-1.50)	0.98 (0.78-1.22)	1.00 (0.51-1.97)	
Male≥90, female≥85	4.7	3.5	5.19 (1.75-15.35)	1.15 (0.34-3.86)	1.03 (0.55-1.95)	1.64 (1.14-2.36)	0.90 (0.59-1.38)	NA[g]	
**Family history of CVD [f]**									0.93
No	4.4	3.0	4.02 (2.17-7.46)	2.71 (1.67-4.38)	1.33 (0.99-1.80)	1.29 (1.04-1.59)	0.91 (0.73-1.14)	0.65 (0.30-1.38)	
Yes	5.0	3.6	2.71 (0.55-13.47)	0.92 (0.27-3.08)	1.09 (0.55-2.13)	1.35 (0.85-2.14)	1.15 (0.75-1.74)	1.33 (0.31-5.75)	

CI, confidence interval; PYs, person-years; COPD: chronic obstructive pulmonary disease; CVD, cardiovascular diseases.

[a] Data were adjusted for age at index date, sex, and study area where appropriate.

[b] Please refer to Figure 2 for covariates adjusted in the models, except in case of stratifying by corresponding variable.

[c] The disease status of hypertension, diabetes, and COPD were updated until the index date.

[d] Participants who quit smoking because of illness were classified as current daily smokers.

[e] Excessive drinker was defined as those who drank ≥30 g of pure alcohol per day, or having stopped drinking.

[f] A CVD family history was defined as having at least one first-degree relative (biological father, mother, and siblings) with heart disease or stroke.

[g] Estimates were not available due to the small number of cases.

Time interval after the index date	Pneumonia cases No. of events (Rate)[a]	Matched controls No. of events (Rate)[a]	Hazard ratio (95% CI) Unadjusted	Adjusted[b]	*P*-value[b]
**MCE**					
0-30 d	38 (19.5)	26 (1.4)	13.69 (8.32-22.55)	11.21 (6.58-19.08)	<0.0001
31-90 d	16 (4.2)	58 (1.6)	2.63 (1.51-4.58)	2.05 (1.15-3.66)	0.01
91 d-1 y	84 (5.2)	282 (1.8)	2.94 (2.31-3.75)	2.50 (1.94-3.22)	<0.0001
(1-2] y	69 (3.9)	372 (2.0)	2.02 (1.56-2.61)	1.87 (1.44-2.44)	<0.0001
(2-3] y	50 (3.5)	378 (2.3)	1.54 (1.15-2.07)	1.32 (0.98-1.79)	0.07
(3-4] y	40 (3.6)	322 (2.3)	1.56 (1.12-2.17)	1.46 (1.05-2.05)	0.03
(4-5] y	34 (4.0)	287 (2.5)	1.59 (1.11-2.26)	1.47 (1.02-2.11)	0.03
(5-8] y	51 (3.7)	620 (3.0)	1.25 (0.94-1.67)	1.27 (0.95-1.70)	0.10
(8-10] y	11 (3.4)	192 (3.5)	0.98 (0.54-1.81)	1.12 (0.60-2.06)	0.73
**IHD other than MCE**					
0-30 d	95 (48.7)	148 (8.1)	6.03 (4.66-7.80)	4.99 (3.81-6.53)	<0.0001
31-90 d	115 (30.5)	297 (8.2)	3.71 (2.99-4.60)	3.33 (2.67-4.16)	<0.0001
91 d-1 y	388 (24.6)	1215 (7.7)	3.19 (2.85-3.58)	2.73 (2.42-3.07)	<0.0001
(1-2] y	409 (24.2)	1643 (8.7)	2.77 (2.48-3.09)	2.41 (2.16-2.70)	<0.0001
(2-3] y	292 (21.8)	1580 (9.7)	2.24 (1.97-2.53)	2.02 (1.78-2.29)	<0.0001
(3-4] y	251 (24.4)	1338 (9.8)	2.48 (2.17-2.84)	2.29 (1.99-2.62)	<0.0001
(4-5] y	164 (21.2)	1114 (10.1)	2.10 (1.78-2.48)	1.96 (1.66-2.32)	<0.0001
(5-8] y	238 (19.6)	2376 (11.9)	1.65 (1.44-1.88)	1.59 (1.39-1.82)	<0.0001
(8-10] y	44 (15.7)	649 (12.5)	1.26 (0.93-1.71)	1.25 (0.92-1.71)	0.15

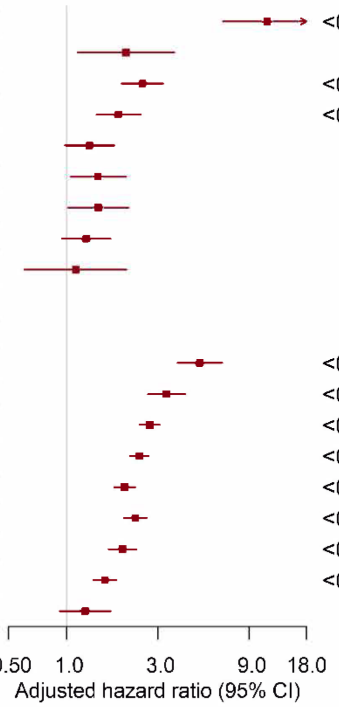

Fig. S1. Risk of ischaemic heart disease by subtypes after hospitalisation for pneumonia

CI, confidence interval; MCE, major coronary events; IHD, ischaemic heart disease.

The area of each square is inversely proportional to the variance, and 95% confidence intervals are shown.

[a] Rates are equal to the number of events per 1000 person-years.

[b] Please refer to Figure 2 for covariates adjusted in the models. *P* values pertain to adjusted hazard ratios.

Time interval after the index date	Pneumonia cases No. of events (Rate)[a]	Matched controls No. of events (Rate)[a]	Hazard ratio (95% CI) Unadjusted	Hazard ratio (95% CI) Adjusted[b]	*P*-value[b]
**LACI**					
0-30 d	8 (4.1)	26 (1.4)	2.89 (1.31-6.37)	2.21 (0.98-4.98)	0.06
31-90 d	11 (2.9)	45 (1.2)	2.33 (1.21-4.51)	2.23 (1.13-4.39)	0.02
91 d-1 y	62 (3.9)	216 (1.4)	2.84 (2.14-3.76)	2.59 (1.94-3.46)	<0.0001
(1-2] y	63 (3.6)	313 (1.7)	2.19 (1.67-2.87)	2.02 (1.53-2.66)	<0.0001
(2-3] y	40 (2.8)	295 (1.8)	1.59 (1.14-2.21)	1.41 (1.01-1.97)	0.05
(3-4] y	35 (3.2)	287 (2.1)	1.53 (1.08-2.18)	1.46 (1.02-2.08)	0.04
(4-5] y	23 (2.7)	275 (2.4)	1.12 (0.73-1.72)	1.05 (0.68-1.61)	0.83
(5-8] y	48 (3.5)	503 (2.4)	1.46 (1.08-1.96)	1.36 (1.01-1.84)	0.04
(8-10] y	12 (3.8)	142 (2.6)	1.47 (0.81-2.64)	1.38 (0.76-2.51)	0.28
**Non-LACI**					
0-30 d	55 (28.2)	177 (9.7)	2.92 (2.16-3.95)	2.62 (1.91-3.59)	<0.0001
31-90 d	81 (21.4)	299 (8.3)	2.59 (2.02-3.31)	2.56 (1.99-3.30)	<0.0001
91 d-1 y	229 (14.4)	1564 (9.9)	1.45 (1.26-1.67)	1.38 (1.19-1.58)	<0.0001
(1-2] y	274 (16.0)	1908 (10.2)	1.57 (1.38-1.78)	1.51 (1.33-1.72)	<0.0001
(2-3] y	244 (17.8)	1756 (10.9)	1.64 (1.43-1.87)	1.61 (1.41-1.85)	<0.0001
(3-4] y	179 (16.9)	1610 (11.9)	1.42 (1.22-1.66)	1.45 (1.24-1.69)	<0.0001
(4-5] y	152 (18.8)	1413 (12.9)	1.46 (1.24-1.73)	1.52 (1.28-1.80)	<0.0001
(5-8] y	207 (16.2)	2782 (14.0)	1.15 (1.00-1.33)	1.24 (1.07-1.43)	0.0033
(8-10] y	41 (13.9)	747 (14.5)	0.96 (0.70-1.31)	1.17 (0.85-1.60)	0.34

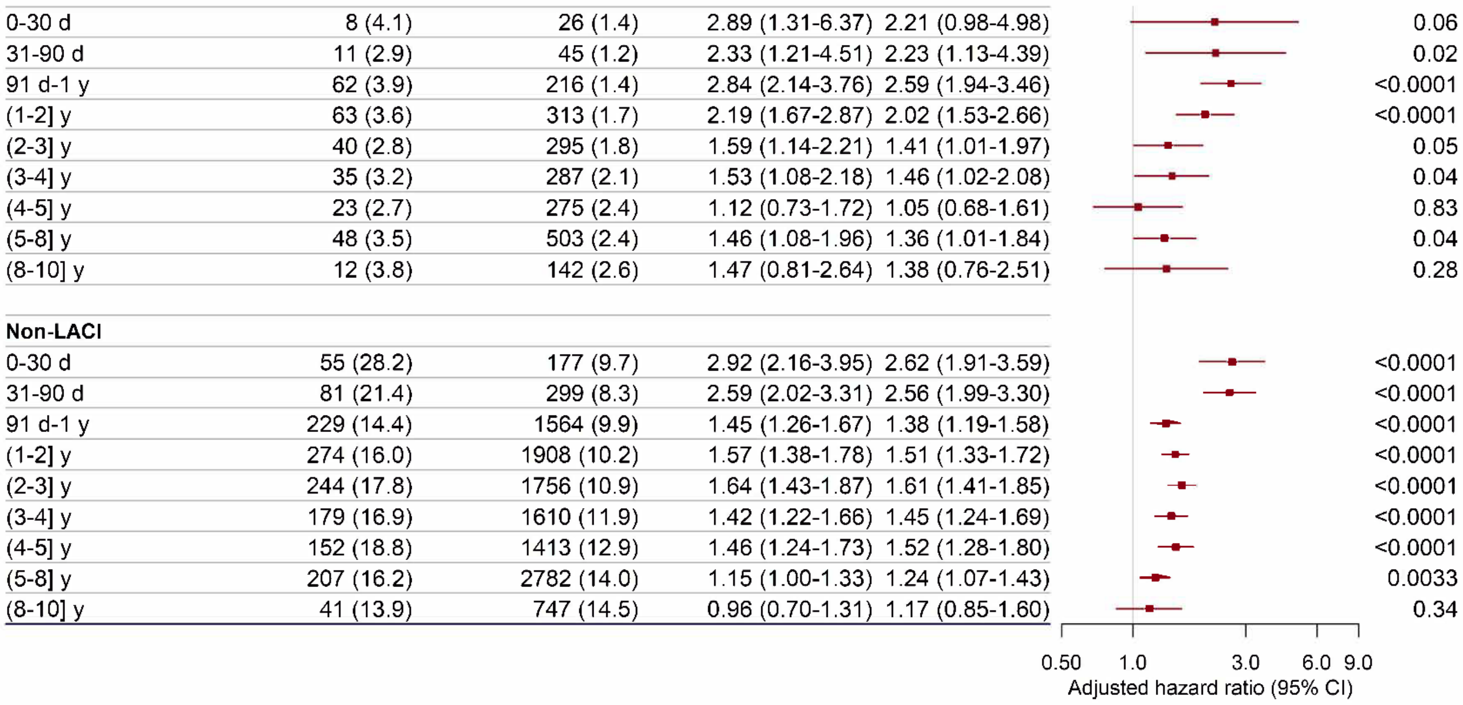

Fig. S2. Risk of ischaemic stroke by subtypes after hospitalisation for pneumonia

CI, confidence interval; LACI, lacunar infarction.

The area of each square is inversely proportional to the variance, and 95% confidence intervals are shown.

[a] Rates are equal to the number of events per 1000 person-years.

[b] Please refer to Figure 2 for covariates adjusted in the models. *P* values pertain to adjusted hazard ratios.
