# Supplementary material for: Association between pneumonia hospitalisation and long-term risk of cardiovascular disease in Chinese adults: A prospective cohort study
Source: eClinicalMedicine. 2022 Dec 2;55:101761. doi: 10.1016/j.eclinm.2022.101761 (PMC9722470; doi:10.1016/j.eclinm.2022.101761)
Supplement: Caption for Supplementary Material [file mmc2.docx]

**Caption for Supplementary Material**

Translated Abstract

Supplementary Methods, Tables S1–S11, Figures S1 and S2

List of All Study Group Members
