# Supplementary material for: Association between pneumonia hospitalisation and long-term risk of cardiovascular disease in Chinese adults: A prospective cohort study
Source: eClinicalMedicine. 2022 Dec 2;55:101761. doi: 10.1016/j.eclinm.2022.101761 (PMC9722470; doi:10.1016/j.eclinm.2022.101761)
Supplement: List of All Study Group Members [file mmc3.docx]

| First and middle names | Surnames |
| --- | --- |
| Junshi | Chen |
| Zhengming | Chen |
| Robert | Clarke |
| Rory | Collins |
| Yu | Guo |
| Liming | Li |
| Jun | Lv |
| Richard | Peto |
| Robin | Walters |
| Daniel | Avery |
| Derrick | Bennett |
| Ruth | Boxall |
| Sue | Burgess |
| Ka Hung | Chan |
| Yumei | Chang |
| Yiping | Chen |
| Johnathan | Clarke |
| Huaidong | Du |
| Ahmed Edris | Mohamed |
| Zammy | Fairhurst-Hunter |
| Hannah | Fry |
| Simon | Gilbert |
| Alex | Hacker |
| Mike | Hill |
| Michael | Holmes |
| Pek Kei | Im |
| Andri | Iona |
| Maria | Kakkoura |
| Christiana | Kartsonaki |
| Rene | Kerosi |
| Kuang | Lin |
| Mohsen | Mazidi |
| Iona | Millwood |
| Sam | Morris |
| Qunhua | Nie |
| Alfred | Pozarickij |
| Paul | Ryder |
| Saredo | Said |
| Sam | Sansome |
| Dan | Schmidt |
| Paul | Sherliker |
| Rajani | Sohoni |
| Becky | Stevens |
| Iain | Turnbull |
| Lin | Wang |
| Neil | Wright |
| Ling | Yang |
| Xiaoming | Yang |
| Pang | Yao |
| Xiao | Han |
| Can | Hou |
| Pei | Pei |
| Chao | Liu |
| Canqing | Yu |
| Qingmei | Xia |
| Zengchang | Pang |
| Ruqin | Gao |
| Shanpeng | Li |
| Haiping | Duan |
| Shaojie | Wang |
| Yongmei | Liu |
| Ranran | Du |
| Yajing | Zang |
| Liang | Cheng |
| Xiaocao | Tian |
| Hua | Zhang |
| Yaoming | Zhai |
| Feng | Ning |
| Xiaohui | Sun |
| Feifei | Li |
| Silu | Lv |
| Junzheng | Wang |
| Wei | Hou |
| Wei | Sun |
| Shichun | Yan |
| Xiaoming | Cui |
| Chi | Wang |
| Zhenyuan | Wu |
| Yanjie | Li |
| Quan | Kang |
| Huiming | Luo |
| Tingting | Ou |
| Xiangyang | Zheng |
| Zhendong | Guo |
| Shukuan | Wu |
| Yilei | Li |
| Huimei | Li |
| Ming | Wu |
| Yonglin | Zhou |
| Jinyi | Zhou |
| Ran | Tao |
| Jie | Yang |
| Jian | Su |
| Fang | Liu |
| Jun | Zhang |
| Yihe | Hu |
| Yan | Lu |
| Liangcai | Ma |
| Aiyu | Tang |
| Shuo | Zhang |
| Jianrong | Jin |
| Jingchao | Liu |
| Mei | Lin |
| Zhenzhen | Lu |
| Lifang | Zhou |
| Changping | Xie |
| Jian | Lan |
| Tingping | Zhu |
| Yun | Liu |
| Liuping | Wei |
| Liyuan | Zhou |
| Ningyu | Chen |
| Yulu | Qin |
| Sisi | Wang |
| Xianping | Wu |
| Ningmei | Zhang |
| Xiaofang | Chen |
| Xiaoyu | Chang |
| Mingqiang | Yuan |
| Xia | Wu |
| Wei | Jiang |
| Jiaqiu | Liu |
| Qiang | Sun |
| Faqing | Chen |
| Xiaolan | Ren |
| Caixia | Dong |
| Hui | Zhang |
| Enke | Mao |
| Xiaoping | Wang |
| Tao | Wang |
| Xi | zhang |
| Kai | Kang |
| Shixian | Feng |
| Huizi | Tian |
| Lei | Fan |
| XiaoLin | Li |
| Huarong | Sun |
| Pan | He |
| Xukui | Zhang |
| Min | Yu |
| Ruying | Hu |
| Hao | Wang |
| Xiaoyi | Zhang |
| Yuan | Cao |
| Kaixu | Xie |
| Lingli | Chen |
| Dun | Shen |
| Xiaojun | Li |
| Donghui | Jin |
| Li | Yin |
| Huilin | Liu |
| Zhongxi | Fu |
| Xin | Xu |
| Hao | Zhang |
| Jianwei | Chen |
| Yuan | Peng |
| Libo | Zhang |
| Chan | Qu |
