# Supplementary material for: Association between pneumonia hospitalisation and long-term risk of cardiovascular disease in Chinese adults: A prospective cohort study
Source: eClinicalMedicine. 2022 Dec 2;55:101761. doi: 10.1016/j.eclinm.2022.101761 (PMC9722470; doi:10.1016/j.eclinm.2022.101761)
Supplement: Translated Abstract [file mmc4.docx]

**中国成人肺炎住院与随后短期和长期心血管疾病风险的关联：一项前瞻性队列研究**

**摘要**

**背景：**既往研究提示，包括肺炎在内的下呼吸道感染与短期（主要是1年内）的心血管疾病发病风险增加有关，但少有研究探究肺炎后更长时间范围内的心血管疾病风险。

**方法：**本研究基于中国慢性病前瞻性研究（China Kadoorie Biobank）于2004年6月25日至2008年7月15日期间募集的482,017名基线和肺炎住院前无心血管疾病的研究对象。从基线调查至2018年12月31日共有24,060名研究对象发生肺炎住院，每个肺炎病例均根据年龄、性别、居住地区和衰弱状态等特征匹配至多15名对照，共223,875名对照纳入分析。我们使用分段Cox比例风险回归模型来估计肺炎住院与随后10年内不同时间区间缺血性心脏病、心律失常、心衰、缺血性卒中和出血性卒中等5种心血管疾病结局发病风险的关联效应值（HR）及其95%的可信区间（CI）。

**结果：**当前的匹配队列研究共纳入247,935名肺炎病例和对照，基线年龄（标准差）为53·5（10·4）岁，其中男性占40·8%（101,159）。在随访过程中，肺炎住院患者随后发生缺血性心脏病、心律失常、心衰、缺血性卒中和出血性卒中的人数（比例）分别为2389（9·9%）、489（2·0%）、545（2·3%）、1764（7·3%）和348（1·4%）。在调整社会经济学特征、生活方式、健康及用药情况、基础疾病和心血管疾病家族史等可能的混杂因素后，肺炎住院后30天内心血管疾病的发病风险最高，随后，不同类型的心血管疾病增加的风险以不同的速度和幅度逐渐降低。对于缺血性心脏病、心律失常和心衰，这种增加的风险直到肺炎住院后的第8年仍显著高于对照组，对应的HRs（95% CIs）分别为1·48（1·13-1·93）、2·69（1·70-4·25）和4·36（2·86-6·64）。肺炎后缺血性卒中的增加的风险持续至住院后的第7年（HR=1·30; 95% CI: 1·04-1·63），出血性卒中增加的风险则持续至住院后的第2年（1·39; 1·07-1·80）。在不同的亚组人群中，我们观察到了类似的关联。

**解读：**在中国的中老年人群中，肺炎住院与增加的短期和长期心血管疾病风险有关，这种增加的风险最长可以持续8年之久。
